# Demethylase-independent function of JMJD2D as a novel antagonist of p53 to promote Liver Cancer initiation and progression

**DOI:** 10.7150/thno.45581

**Published:** 2020-07-11

**Authors:** Ming Li, Yuan Deng, Minghui Zhuo, Hui Zhou, Xu Kong, Xiaogang Xia, Zhaojie Su, Qiang Chen, Peng Guo, Pingli Mo, Chundong Yu, Wengang Li

**Affiliations:** 1 Department of Hepatobiliary Surgery, Xiang'an Hospital of Xiamen University, School of Medicine, Xiamen University, China.; 2 State Key Laboratory of Cellular Stress Biology, Innovation Center for Cell Biology, School of Life Sciences, Xiamen University, Xiamen, China.

**Keywords:** liver cancer, JMJD2D, p53, p21, PUMA

## Abstract

**Background:** As a histone demethylase, JMJD2D can enhance gene expression by specifically demethylating H3K9me2/3 and plays an important role in promoting colorectal cancer progression. However, its role in liver cancer remains unclear.

**Methods:** The expression of JMJD2D was examined in human liver cancer specimens and non-tumorous liver tissues by immunohistochemical or immunoblot analysis. JMJD2D expression was knocked down in liver cancer cells using small hairpin RNAs, and cells were analyzed with Western blot, real-time PCR, cell viability, colony formation, and flow cytometry assays. Cells were also grown as tumor xenografts in nude mice, and the tumor cell proliferation and apoptosis were measured by immunohistochemical analysis. The relationship between JMJD2D and p53 was studied by co-immunoprecipitation, chromatin immunoprecipitation, and electric mobility shift assay. Wild-type and JMJD2D-knockout mice were intraperitoneally injected with diethylnitrosamine (DEN) to induce liver tumors and the liver cancer initiation and progression were investigated.

**Results:** JMJD2D was frequently upregulated in human liver cancer specimens compared with non-tumorous liver tissues. The overall survival of liver cancer patients with high JMJD2D expression was significantly decreased compared to that with low JMJD2D expression. JMJD2D knockdown reduced liver cancer cell proliferation and xenograft tumor growth, sensitized cells to chemotherapeutic drug-induced apoptosis, and increased the expression of cell cycle inhibitor p21 and pro-apoptosis gene PUMA. Genetically, JMJD2D deficiency protected mice against DEN-induced liver cancer initiation and progression. Knockout of tumor suppressor p53 significantly reduced the effects of JMJD2D knockdown on cell proliferation, apoptosis, and the expression of p21 and PUMA, suggesting that JMJD2D regulates liver cancer cell functions in part through inhibiting p53 signaling pathway. Mechanistically, JMJD2D directly interacted with p53 and inhibited p53 recruitment to the p21 and PUMA promoters in a demethylation activity-independent manner, implicating a demethylase-independent function of JMJD2D as a novel p53 antagonist. In addition, JMJD2D could activate Wnt/β-catenin signaling to promote liver cancer cell proliferation.

**Conclusion:** Our study demonstrates that JMJD2D can antagonize the tumor suppressor p53 and activate an oncogenic signaling pathway (such as Wnt/β-catenin signaling pathway) simultaneously to promote liver cancer initiation and progression, suggesting that JMJD2D may serve as a novel target for liver cancer treatment.

## Introduction

Primary liver cancer is the fifth most common cancer worldwide and the fourth highest cause of cancer-related death 1. Hepatocellular carcinoma (HCC), the most common primary malignant liver tumor, grows rapidly and frequently associates with poor prognosis and is projected to become the third leading cause of cancer-related death worldwide by 2020 2. Despite current developments in clinical therapies and research, the 5-year survival rate for liver cancer has not been improved well 3. Therefore, it is imperative to identify potential biological markers and define the molecular mechanisms of liver cancer development for improving clinical diagnosis and therapies.

The tumor suppressor and transcription factor p53 is the most commonly silenced or mutated gene in cancer 4. The most noticeably biological outcomes of p53 activation are cell-cycle arrest and apoptosis, which play important roles in the inhibition of tumor progression 5, 6. Extensive evidence has shown that p53 induces G1/S boundary cell cycle arrest and apoptosis largely (albeit not exclusively) through direct transcriptional induction of CDK inhibitor p21 and puma/noxa, respectively 7.

Histone lysine demethylases (KDMs) belong to JMJD (Jumonji C domain-containing) protein family, which comprises approximate 30 members depending on the presence of other domains and the homological degree of the shared JMJC domain 8. JMJD proteins are involved in normal developments and diseases such as cancer largely through the regulation of chromatin structure and transcription 8. JMJD2D (KDM4D) belongs to the JMJD2 subfamily (including 4 members, JMJD2A-D), which catalyses demethylation of lysine 9 on histone 3 (H3K9) and lysine 26 on histone 1.4 (H1.4K26) 9. JMJD2D can regulate DNA replication and DNA damage repair 10, 11, implicating a potential role of JMJD2D in cancer progression. Recent studies show that JMJD2D is highly expressed in gastrointestinal stromal cancer and colorectal cancer and promotes the progression of these cancers 12, 13. However, the expression profile of JMD2D protein in liver cancer and the role of JMJD2D in liver tumorigenesis remain unknown.

In the present study, we showed that JMJD2D was frequently overexpressed in human HCC specimens, JMJD2D promoted liver cancer initiation and progression through inhibition of p53 signaling pathway.

## Material and Methods

### Patients and Liver Tissue Sample

Eighty pairs of HCC specimens and adjacent non-cancer paraffin tissue sections (HLivH160CS01) were purchased from Shanghai Outdo Biotech (Shanghai, China). The immunohistichemical signal of JMJD2D was predominantly located in the nucleus. The immunoreactive score (0-12) is the product of multiplying the positive-cell-proportion score (0-4) and the staining-intensity score (0-3) as previously described 13. The JMJD2D antibody (Catalogue NO. ABE 499) used for immunohistichemistry analysis was purchased from Merck Millipore. Twenty-two pairs of human HCC specimens and adjacent non-cancer tissues used for Western blot analysis were obtained from the Chenggong Hospital of Xiamen University. This study protocol conformed to ethical guidelines and was approved by the Institute Research Ethics Committee at Xiamen University.

### Cell Culture

Liver cancer cell lines HepG2, SK-Hep1, and Huh-7 were cultured in high glucose DMEM (HyClone) supplemented with 10% FBS and penicillin-streptomycin and were maintained in the humidified incubator with 95% air and 5% CO_2_ at 37 °C.

### Diethylnitrosamine (DEN)-induced liver tumor formation

JMJD2D-deficient mice on the C57BL/6 background were generated by TALEN technique as previously described 13. Fifteen day-old male wide-type and JMJD2D-knockout mice were intraperitoneally injected with DEN (25 mg/kg). Mice were sacrificed, and livers were harvested for photographing, quantitation of tumor formation, and immunohistochemical analysis at eight months post-DEN injection. All experimental protocols involving animals were approved by the Institutional Animal Care and Use Committee of Laboratory Animal Center of Xiamen University.

### Real-time PCR

Briefly, total RNA was isolated with trizol reagent (Invitrogen) according to the manufacturer's instructions. Reverse transcription and real-time PCR reactions were performed using Revertra Ace qPCR RT master mix (TOYOBO) and Faststart universal SYBR green master (Roche), respectively. Relative quantification was achieved by normalization to the amount of β-actin. The primers used for real-time PCR are listed as follow: p21 (5'-CAGGGGAGCAGGCTGAAG-3' and 5'-GGATTAGGGCTTCTCTTGG-3'); PUMA (5'-CGACCTCAACGCACAGTACGA-3' and 5'-AGGCACCTAATTGGGCTCCAT-3'); c-Myc (5'-GCTGCTTAGACGCTGGATTT-3' and CACCGAGTCGTAGTCGAGGT); β-actin (5'-AGCGAGCATATCCCCCAAAGTT-3' and 5'-GGGCACGAAGGCTCATCATT-3'); NOXA (5'-AAGGCGCGCAAGAACGCTCA-3' and 5'-GGAAGTTCAGTTTGTCTCCA-3'); GADD45a (5'-AGAGCAGAAGACCGAAAGGATG-3' and 5'-CCAGCAGGCACAACACCAC-3'); Sestrin2 (5'-ATCGTGGCGGACTCCGAGT-3'and5'-TGCTGCTCGAGGCTCTCAG-3').

### Western blot analysis

Western blot analysis was performed as previously described^14^. The antibody used for Western blot analysis are listed as follow: JMJD2D (ab93694; Abcam); p21 (#2947; Cell Signaling Technology); PUMA (#12450; Cell Signaling Technology, ab9643; Abcam); p53 (sc-126; Santa Cruz Biotechnology); p53 (ab131442; Abcam); c-Myc (ab32072; Abcam); PARP (#9532; Cell Signaling Technology); β-catenin (sc7963; Santa Cruz Biotechnology); β-actin (A5441; Sigma Aldrich); Tubulin (#2148; Cell Signaling Technology); POLD1/DNA polymerase δ (K003912P; Solarbio); Cdc45 (A01367-1; Boster).

### Immunohistochemistry

Immunohistichemistry analysis was performed as previously described^14^. The primary antibodies used for immunohistochemistry analysis are listed as follows: Ki67 (ab15580; Abcam); Clvd Caspase-3 (#9661; Cell Signaling Technology); p21 (ab188224; Abcam); PUMA (sc377015; Santa Cruz Biotechnology); p-p53 (Ser15) (#9284; Cell Signaling Technology).

### Chromatin immunoprecipitation (ChIP) assay

Chromatin immunoprecipitation assay was performed as previously described^14^. Briefly, cells were fixed with 1% formalin for 10 min, and then chromatin was immunoprecipitated using an antibody for p53(sc-6243; Santa Cruz Biotechnology). The ChIP DNA was isolated and detected by quantitative real-time PCR using specific p21 promoter primers (5'-TCCCTATGCTGCCTGCTTCC-3' and 5'-CCACCAGCCTCTTCTATGCC-3'), PUMA promoter primers (5'-CTTTGTGGACCCTGGAACG-3' and 5'-TAGCCCAAGGCAAGGAGG-3').

### Tumor xenograft experiments

5-week-old male nude mice were obtained from the Laboratory Animal Center of Xiamen University. Nude mice were injected subcutaneously in both flanks with 1×10^6^ shCtrl and JMJD2D-knockdown HepG2 cells, respectively. The volume of the tumor was monitored and calculated following the formula: Volume= Length ×Width^2^×0.52. At the end of this experiment, tumors were harvested and weighed, and then were dissected for fixing and embedding in paraffin. All experimental protocols involving animals were approved by the Institutional Animal Care and Use Committee of Laboratory Animal Center of Xiamen University.

### Electric mobility shift assay

Biotin end-labeled or unlabeled sense and antisense oligonucleotides were synthesized and annealed to generate the double-stranded DNA probes. The probe sequences were listed as follows: The p53 binding element on the p21 promoter: 5'-CTGGCCGTCAGGAACATGTCCCAACATGTTGAGCTCT-3'; the mutated p53 binding element on the p21 promoter: 5'-CTGGCCGTCA GGAATATATCCCAATATATTGAGCTCT-3'; the p53 binding element on the PUMA promoter: 5'-GCGCGCCTGCAAGTCCTGACTTGTCCGCGGCG-3'; the mutated p53 binding element on the PUMA promoter: 5'-GCGCGCCTGTAAATCCTGATTTATCCGCGGCG-3'. Binding assays were performed in a buffer containing 5 mM Hepes (PH 7.6), 100 mM KCl, 5 mM MgCl_2_, 1.25 mM DTT, 0.05% NP-40, 2.25% glycerol, 1 μg Poly (dI.dC). Recombinant human p53 was purchased from Active Motif (Catalog No: 81091) and 8 ng of recombinant p53 was used per reaction. E.*coli* extract-based cell-free *in vitro* expression of JMJD2D or JMJD2D-S200M was performed by using the S30 T7 high-yield protein expression system (L1110, Promega). Anti-p53 antibody (OP03, Merck Millipore) was used for super-shift assay. DNA/protein complexes were resolved in a 6% of polyacrylamide gel and analyzed according to the Lightshift chemiluminescent EMSA kit (89880, ThermoFisher).

### Cell death assay

The cell death assay was analyzed by propidiumiodide (PI) staining, as previously described 15. Briefly, cells were resuspended in 1 ml PBS containing 5 μg PI. PI incorporation and cell size were quantified by flow cytometry. Cells were divided into three groups: PI-negative cells with normal size were considered as viable cells; PI-positive cells with smaller size were considered as apoptotic cells of early phase; PI-negative cells with smaller size were considered as dead cells of later period.

### Statistical analysis

All data were shown as the mean+SD of at least three replicates. The statistically significant effects between mean values (p <0.05) were assessed with the two-tailed Student's t-test in SPSS.

## Results

### JMJD2D expression is frequently upregulated in human HCC tissues

**Figure 1:**
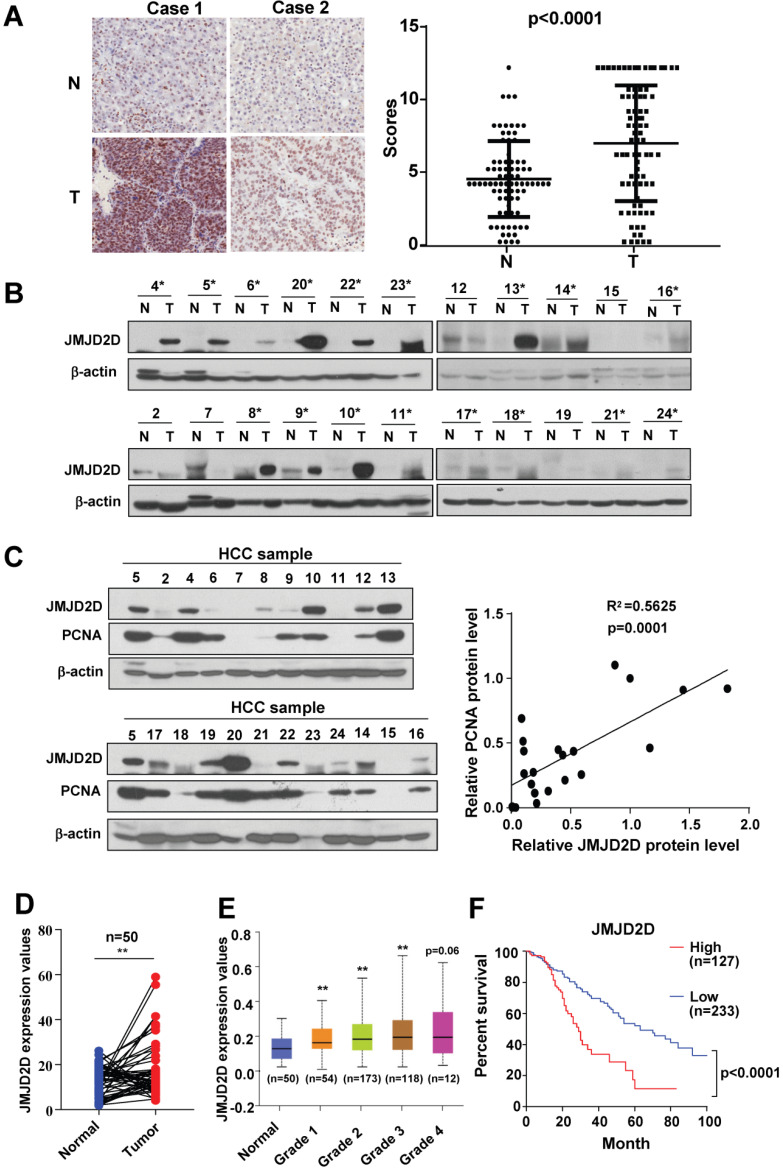
** JMJD2D expression is frequently upregulated in HCC tissues.** (**A**) JMJD2D protein levels in 80 paired human HCC specimens and the surrounding non-tumour tissues were measured and scored by immunohistochemical analysis. (**B**) JMJD2D protein levels in 22 paired human HCC specimens and the surrounding non-tumor tissues were measured by western blot analysis. (**C**) The expression of JMJD2D and PCNA were positively correlated. JMJD2D and PCNA protein levels in 22 human HCC specimens were measured and quantified by western blot analysis. Correlation between JMJD2D protein levels and PCNA protein levels were analyzed. (**D**) JMJD2D mRNA expression was frequently overexpressed in 50 pairs of liver cancer specimens in TCGA database. *p<0.5, **p<0.01. (**E**) JMJD2D expression levels were significantly elevated as early as grade I liver cancer development stage in UALCAN database. *p<0.5, **p<0.01. (**F**) The overall survival in liver cancer patients with high JMJD2D expression were significantly decreased compared with low JMJD2D expression in oncoLnc database. p<0.0001. The data of JMJD2D mRNA expression in 360 liver cancer patients were downloaded from oncoLnc database. JMJD2D expression above average was defined as high expression group (127), whereas JMJD2D expression below average was defined as low expression group (233).

To examine the protein expression profile of JMJD2D in human HCC specimens and the matched surrounding non-tumorous liver tissues, we performed immunohistochemical analysis to measure the protein levels of JMJD2D in 80 pairs of HCC and adjacent non-tumorous paraffin tissue sections. As shown in Figure 1A and S1, JMJD2D was upregulated in HCC specimens compared with non-tumorous liver tissues. To confirm this finding, we assessed JMJD2D protein expression in a set of 22 human HCC specimens using Western blot analysis. As shown in Figure 1B, elevated JMJD2D protein expression was observed in 17 of 22 (77%) human HCC specimens compared with the surrounding non-tumorous tissues. Furthermore, a positive correlation was identified between the protein levels of JMJD2D and proliferation marker proliferating cell nuclear antigen (PCNA) (Figure 1C). Consistently, TCGA data showed that the mRNA levels of JMJD2D in 50 human liver cancer specimens were remarkably increased compared with paired normal liver tissues (Figure 1D). JMJD2D levels in another cohort of human liver cancer specimens were significantly elevated as early as grade I liver cancer development stage in UALCAN database (Figure 1E). The overall survival rate of liver cancer patients with high JMJD2D expression was significantly reduced compared with that with low JMJD2D expression in oncoLnc database (Figure 1F). Collectively, these results suggest that JMJD2D upregulation may promote liver cancer progression.

### Downregulation of JMJD2D inhibits liver cancer cell proliferation and xenograft tumor growth

**Figure 2:**
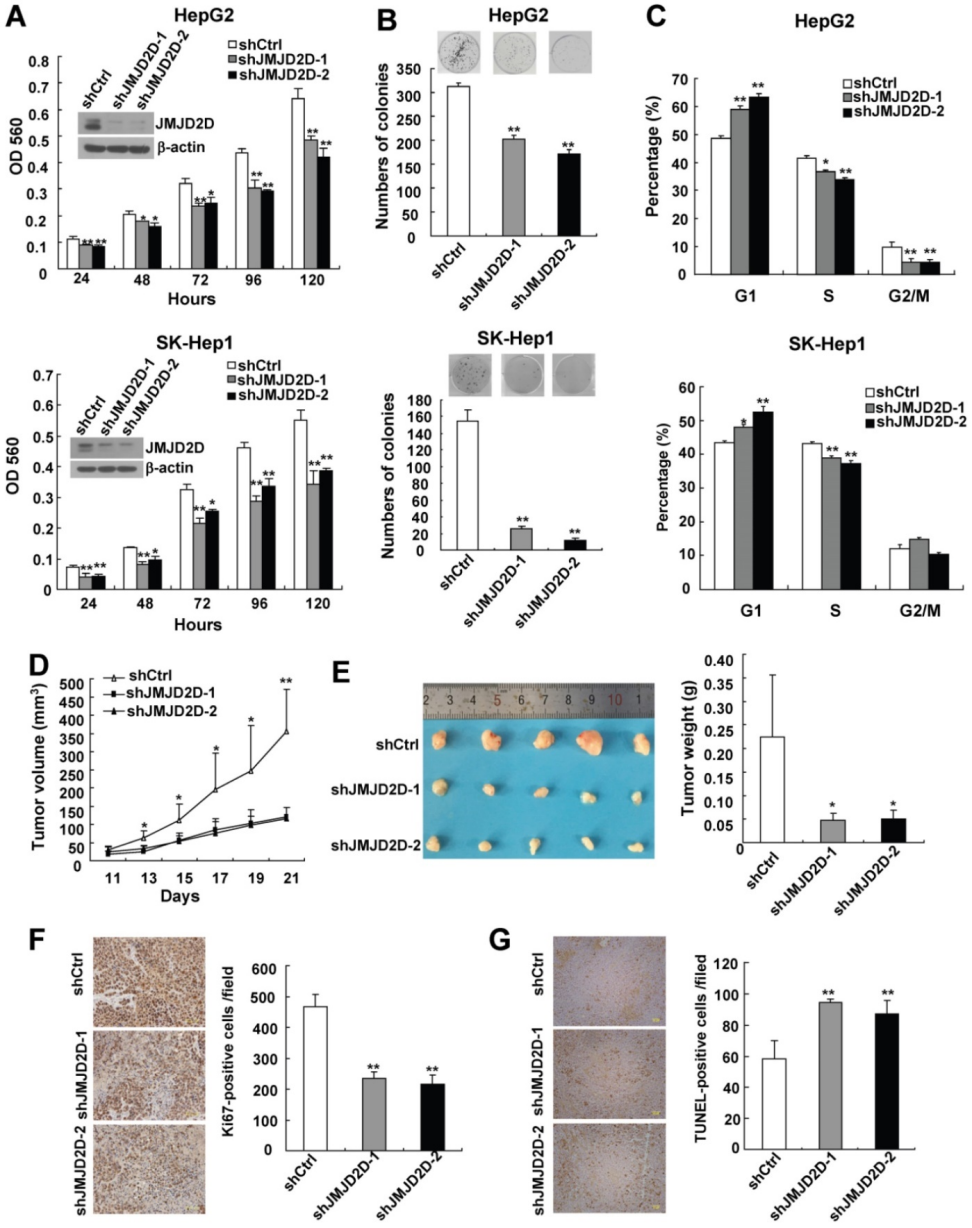
** Downregulation of JMJD2D inhibits liver cancer cell proliferation and xenograft tumor growth.** (**A**) Knockdown of JMJD2D inhibited HepG2 and SK-Hep1 cell proliferation, as measured by MTT assay. (**B**) Knockdown of JMJD2D inhibited the ability of clone formation in HepG2 and SK-Hep1 cells, as measured by clone formation assay. (**C**) Knockdown of JMJD2D induced cell arrest in G1 phase of the cell cycle in HepG2 and SK-Hep1 cells, as measured by flow cytometric analysis. (**D-E**) Knockdown of JMJD2D inhibited HepG2 xenograft tumor growth and decreased tumor weight. (**F**) Knockdown of JMJD2D inhibited cell proliferation of HepG2 xenograft tumor, as measured by Ki-67 staining. (**G**)Knockdown of JMJD2D promoted cell apoptosis of HepG2 xenograft tumor, as measured by TUNEL assay. n≥3, *p<0.5, **p<0.01. These experiments were performed at least twice with similar results.

To investigate the role of JMJD2D in liver cancer cell proliferation, two different short hairpin RNA against JMJD2D were used to knock down JMJD2D expression in two human liver cancer cell lines HepG2 and SK-Hep1. MTT and colony formation assays were performed to detect proliferation and colony formation ability of control and JMJD2D-knockdown liver cancer cells. As shown in Figure 2A and 2B, knockdown of JMJD2D decreased cell proliferation and impaired colony formation.

As knockdown of JMJD2D resulted in a decrease in liver cancer cell proliferation, flow cytometric analysis was performed to examine the effect of JMJD2D knockdown on cell cycle progression. As shown in Figure 2C, the percentage of JMJD2D knockdown cells at G1 phase was increased compared with control cells, and this was associated with a concomitant decrease of cells at the S or G2/M phase of the cell cycle, suggesting that downregulation of JMJD2D induces liver cancer cell arrest in G1 phase of the cell cycle, which is at least in part responsible for the suppression of cell proliferation by JMJD2D knockdown.

Having demonstrated that JMJD2D is required for cell proliferation *in vitro*, we further determined whether JMJD2D affects xenograft tumor growth* in vivo*. Control and JMJD2D-knockdown HepG2 cells were subcutaneously injected into nude mice, and then the tumor sizes were monitored every 2 days from day 11 after cell injection. As shown in Figure 2D, JMJD2D-knockdown liver tumors grew much slower than control tumors. At the end of the study (day 21), tumor weight of JMJD2D-knockdown group was much less than that of the control group (Figure 2E). As expected, Western blot results showed that the protein levels of JMJD2D in JMJD2D-knockdown tumors were much lower than those in control tumors (Figure S2). These results suggest that downregulation of JMJD2D in liver cancer cells inhibits xenograft tumor growth in nude mice.

Furthermore, we performed Ki67 staining and TUNEL assay to examine the effects of JMJD2D knockdown on cell proliferation and apoptosis *in vivo*, respectively. As shown in Figure 2F and 2G, knockdown of JMJD2D significantly decreased Ki67-positive cell number, whereas it increased TUNEL-positive cell number in the tissue sections from JMJD2D-knockdown tumors, indicating that downregulation of JMJD2D inhibits liver cancer cell proliferation but promotes liver cancer cell apoptosis *in vivo*. Collectively, these results demonstrate that JMJD2D promotes xenograft liver tumor growth at least in part through regulating tumor cell proliferation and apoptosis, suggesting that JMJD2D plays an essential promoting role in liver cancer progression.

Interestingly, compensatory upregulation of JMJD2A, JMJD2B, and JMJD2C proteins was detected in JMJD2D-knockdown HepG2 cells (Figure S3). Although the existence of compensatory upregulation of other members of JMJD2 family, knockdown of JMJD2D still strongly inhibited liver cancer progression, suggesting that JMJD2D plays a major role in the development of liver cancer among JMJD2 family members.

### Downregulation of JMJD2D sensitizes liver cancer cells to apoptosis induced by chemotherapeutic drugs

Cell proliferation and resisting cell death are interrelated processes in tumor development.

**Figure 3:**
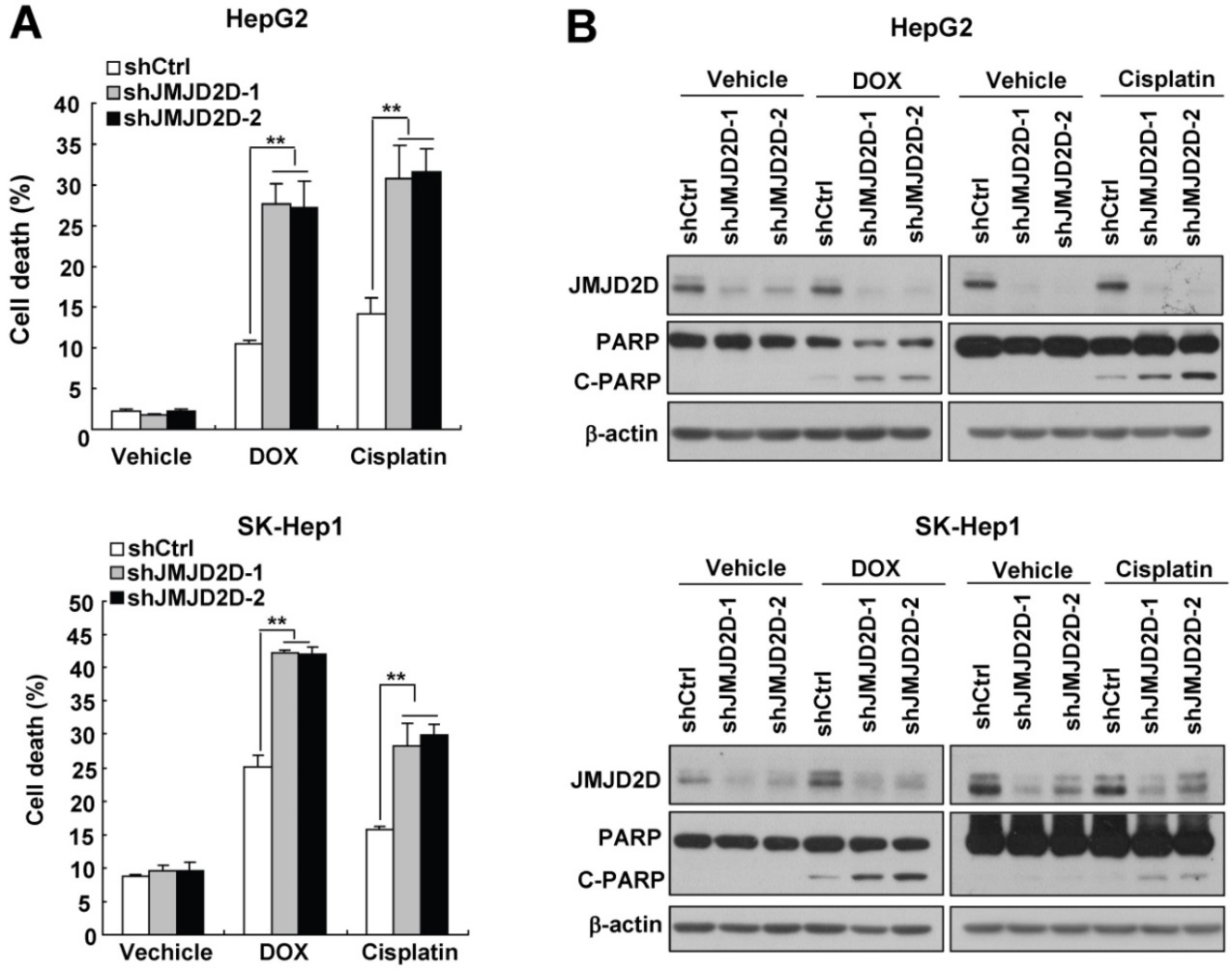
** Downregulation of JMJD2D sensitizes liver cancer cells to apoptosis induced by chemotherapeutic drugs.** (**A**) Downregulation of JMJD2D promoted liver cancer cell death after treatment with chemotherapeutic drugs. Control and JMJD2D-knockdown cells were treated with 0.5 μM doxorubicin or 10 μM cisplatin for 48 hours, then cell death were measured by flow cytometry analysis. n≥3, *p<0.5, **p<0.01. (**B**) Downregulation of JMJD2D promoted liver cancer cell apoptosis after treatment with chemotherapeutic drugs. Control and JMJD2D-knockdown cells were treated with 0.5 μM doxorubicin or 10 μM cisplatin for 48 hours, then the related protein level were measured by western blot. These experiments were performed at least three times with similar results.

Tumor cells evolve a variety of strategies to limit apoptosis in the face of a variety of stresses during tumor formation and cancer therapy. Because liver cancer cells have only a slight apoptotic rate under normal culture condition, to examine the effect of JMJD2D on cell apoptosis, we chose two widely used chemotherapeutic drugs doxorubicin and cisplatin to induce liver cancer cell apoptosis. As shown in Figure 3A, knockdown of JMJD2D in HepG2 and SK-Hep1 cells significantly increased doxorubicin- and cisplatin-induced cell death, respectively. Consistently, Western blot results showed that JMJD2D knockdown enhanced the cleavage of PARP (poly ADP-ribose polymerase, C-PARP), a marker of cell apoptosis, in liver cancer cells treated with chemotherapeutic drugs (Figure 3B). These results suggest that downregulation of JMJD2D sensitizes liver cancer cells to apoptosis induced by chemotherapeutic drugs.

### JMJD2D knockdown-mediated upregulation of p21 and PUMA largely depends on the tumor suppressor p53 in liver cancer cells

**Figure 4:**
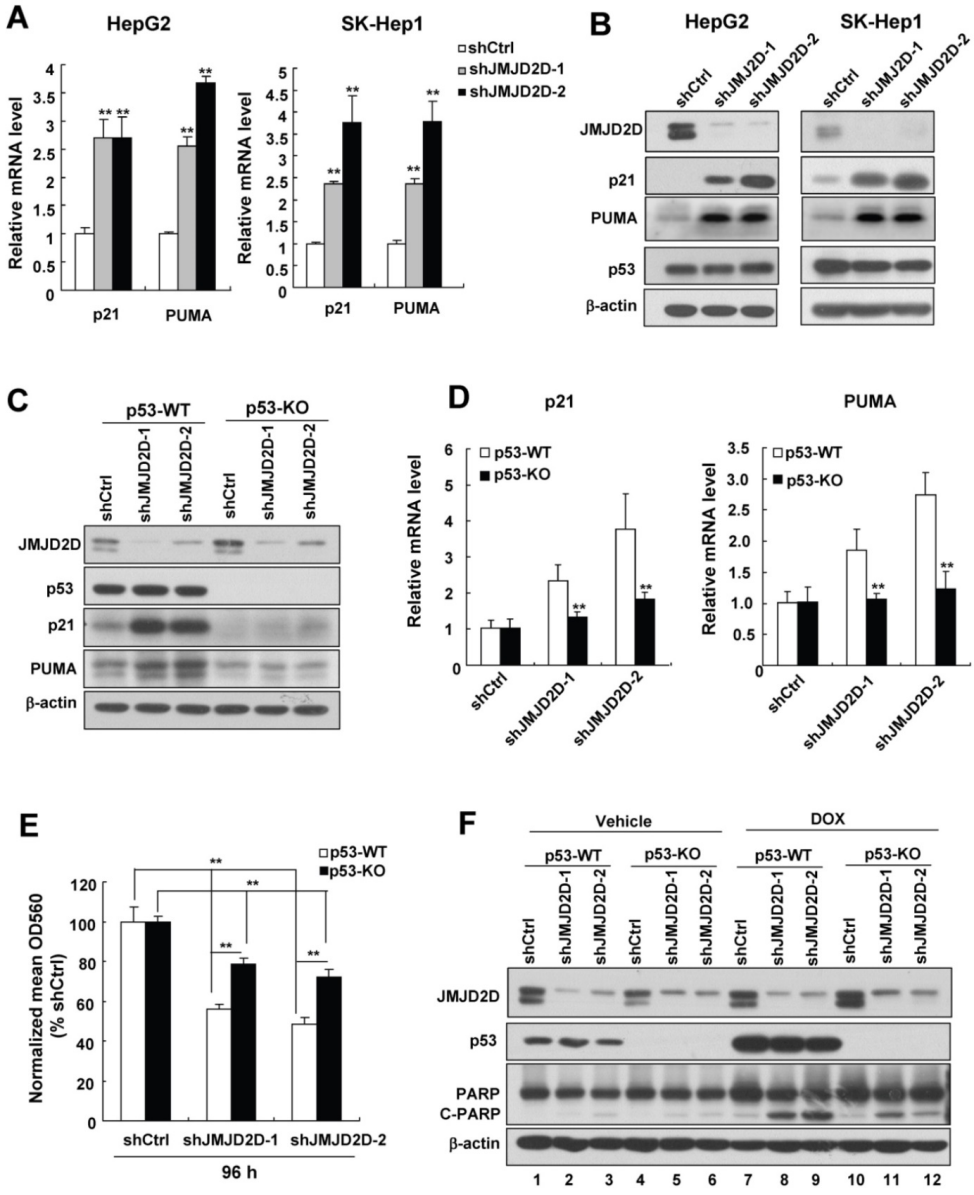
** JMJD2D knockdown-mediated upregulation of p21 and PUMA largely depends on p53 in liver cancer cells.** (**A-B**) Knockdown of JMJD2D increased the mRNA and protein levels of p21 and PUMA in HepG2 and SK-Hep1 cells, as measured by real-time PCR and Western blot, respectively. (**C-D**) p53 knockout dramatically reduced the upregulation effect of JMJD2D knockdown on the expression of p21 and PUMA protein and mRNA in liver cancer cells. The protein and mRNA expression levels of p21 and puma were examined in wild-type and p53^-/-^ SK-Hep1 cells, respectively. (**E-F**) Knockout of p53 significantly reduced the effect of JMJD2D knockdown on cell proliferation and cell apoptosis. Cell proliferation and apoptosis were examined in wild-type and p53^-/-^ SK-Hep1 cells, respectively, as measured by MTT assay and expression of C-PARP, respectively. n≥3, *p<0.5, **p<0.01. These experiments were performed at least three times with similar results.

To investigate the molecular mechanisms by which JMJD2D affects liver cancer cell proliferation and apoptosis, we performed real-time PCR to examine the effects of JMJD2D on the expression of some classic cell proliferation and apoptosis-related genes. As shown in Figure 4A, knockdown of JMJD2D significantly increased the mRNA levels of cell cycle inhibitor p21 and pro-apoptosis gene PUMA. Consistently, Western blot results confirmed that knockdown of JMJD2D upregulated p21 and PUMA protein expression (Figure 4B). Furthermore, we found that JMJD2D expression was inversely correlated with PUMA in human liver cancer specimens from publicly available TCGA data (Figure S4A). However, no correlation was established between the expression levels of JMJD2D and p21 in the same data set (Figure S4B), perhaps due to the fact that other factors being able to regulate p21 expression may interfere with the correlation between JMJD2D and p21. These results suggest that JMJD2D knockdown-mediated upregulation of p21 and PUMA may be at least in part responsible for the effects of JMJD2D knockdown on liver cancer cell proliferation and apoptosis.

Given that p21 and PUMA are direct target genes of tumor suppressor p53 5, and HepG2 and SK-Hep1 cells contain wild-type p53 protein (The IARC TP53 Database) whose levels were not affected by JMJD2D knockdown (Figure 4B), we speculated that downregulation of JMJD2D promotes p21 and PUMA expression at the transcriptional level through activating p53 signaling without affecting p53 protein levels. To test whether regulation of p21 and PUMA gene expression by JMJD2D depends on the p53 signaling pathway, we established stable p53-knockout SK-Hep1 cells using CRISPR-Cas9 system and then examined the effects of JMJD2D on the expression of these genes in p53 wild-type (WT) and knockout (KO) SK-Hep1 cells, respectively. As shown in Figure 4C and 4D, p53 knockout dramatically reduced (even abolished) the upregulation effects of JMJD2D knockdown on the expression of p21 and PUMA protein and mRNA in liver cancer cells, indicating that JMJD2D knockdown-mediated upregulation of p21 and PUMA expression largely depends on p53. Knockdown of JMJD2D did not affect the expression of p21 and PUMA in human liver cancer Huh-7 cells (Figure S5), which express the p53-Y220C mutant with impaired transactivation activity 16-18, supporting the notion that the effect of JMJD2D on p21 and PUMA expression largely depends on a functional p53.

To determine whether JMJD2D regulates cell proliferation and apoptosis depending on p53 signaling pathway, we examined the effects of JMJD2D knockdown on cell proliferation and apoptosis in p53-WT and p53-KO SK-Hep1 cells, respectively. As shown in Figure 4E and 4F, knockout of p53 significantly reduced the effects of JMJD2D knockdown on cell proliferation and apoptosis, respectively, suggesting that JMJD2D regulates cell proliferation and apoptosis in part through p53 signaling pathway.

### JMJD2D directly interacts with p53 and inhibits p53 recruitment to the p21 and PUMA gene promoters

**Figure 5:**
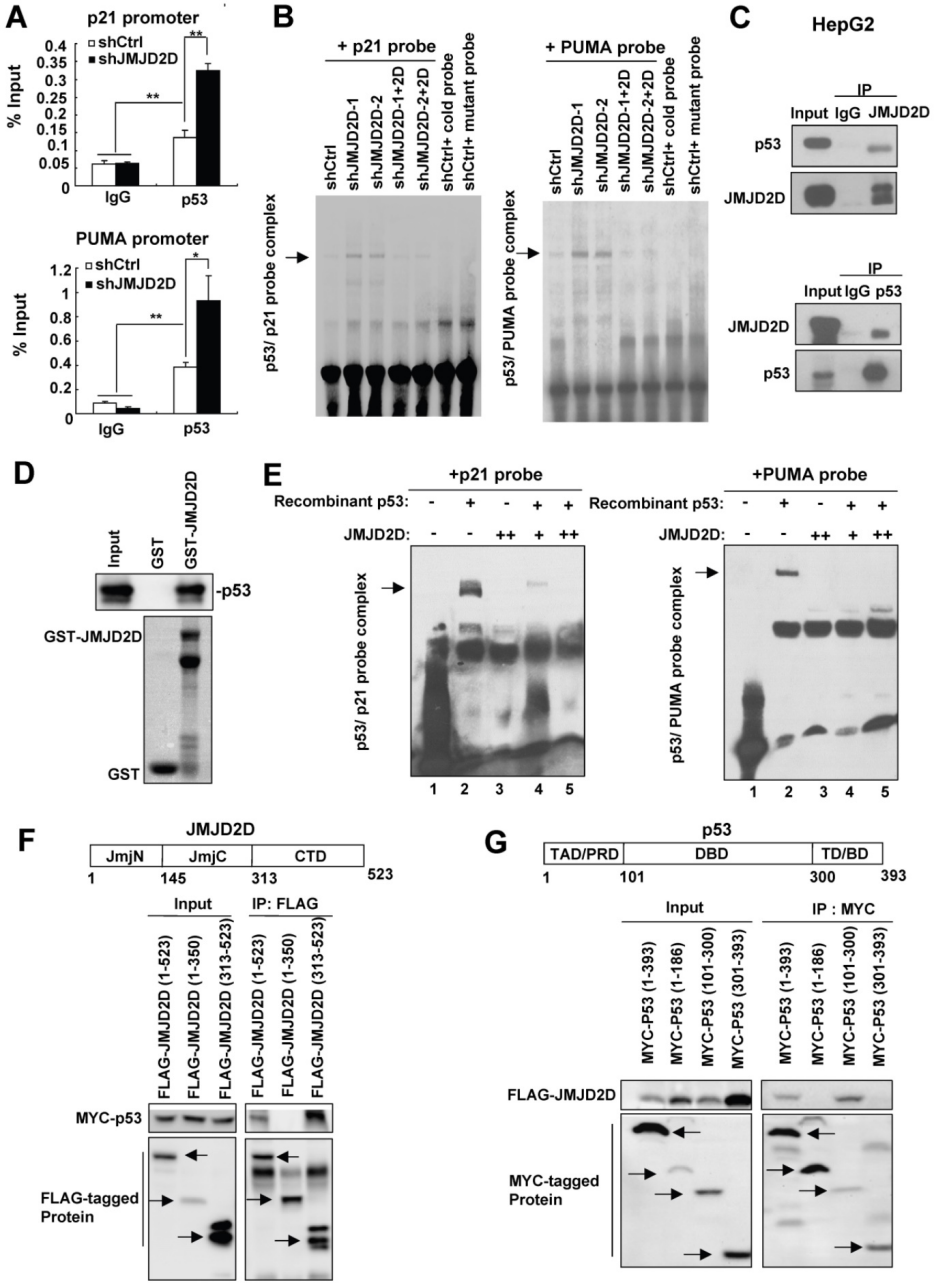
** JMJD2D directly interacts with p53 and inhibits p53 recruitment on the p21 or PUMA gene promoter.** (**A**) Knockdown of JMJD2D enhanced p53 recruitment at p53 binding sites on the p21 and PUMA promoters, as measured by chromatin immunoprecipitation assay. n≥3, *p<0.5, **p<0.01. (**B**) Knockdown of JMJD2D enhanced p53 binding to p21 and PUMA probes in SK-Hep1 cells, whereas restoration of JMJD2D expression in JMJD2D-knockdown cells inhibited p53 binding to the p21 and PUMA probes, as measured by EMSA assay. (**C**) Endogenous JMJD2D interacted with p53 in HepG2 cells, as measured by Co-IP assay. (**D**) GST pull-down analysis of the interaction between JMJD2D and p53 in vitro. E. *coli*-produced GST or GST-JMJD2D protein was incubated with recombinant human p53 protein. (**E**) JMJD2D protein produced by an E. *coli* extract-based cell-free protein synthesis system physically blocked p53 binding to p21 and PUMA probes, as measured by EMSA assay *in vitro*. (**F**) Co-IP assay analysis of the interaction between p53 and different domains of JMJD2D. (**G**) Co-IP assay analysis of the interaction between JMJD2D and different domains of p53. These experiments were performed at least three times with similar results.

Having demonstrated that JMJD2D knockdown-mediated upregulation of p21 and PUMA largely depends on p53, we further pursued the underlying mechanism. Knockdown of JMJD2D did not affect p53 protein levels in both cytoplasm and nucleus (Figures S6), suggesting that JMJD2D knockdown does not affect the nuclear translocation of p53 proteins. Therefore, JMJD2D knockdown may affect the DNA binding activity or the transcriptional activity of p53 protein. To determine whether JMJD2D affects p53 binding to p21 or PUMA promoter, the chromatin immunoprecipitation (ChIP) assay was performed. As shown in Figure 5A, JMJD2D knockdown enhanced p53 recruitment to the p53 binding sites on the p21 and PUMA promoters, respectively. Furthermore, the influence of JMJD2D knockdown on p53 binding to p53 response element (PRE) on the p21 and PUMA promoters was determined by an electrophoretic mobility shift assay (EMSA) with labeled p21 and PUMA oligonucleotide probes, respectively. As shown in Figure 5B and Figure S7, p53 binding to p21 and PUMA probes was significantly increased in JMJD2D-knockdown cells compared with control cells, whereas restoration of JMJD2D expression in JMJD2D-knockdown cells could inhibit p53 binding to the p21 and PUMA probes, suggesting that JMJD2D affects the DNA binding activity of p53 protein. Consistently, restoration of JMJD2D expression in JMJD2D-knockdown cells dramatically reduced the upregulation effects of JMJD2D knockdown on the expression of p21 and PUMA protein (Figure S8)**.** Collectively, these results implicate that JMJD2D inhibits the transcriptional function of p53 by blocking p53 binding to target gene to consequently attenuate the expression of p21 or PUMA at the transcriptional level.

To examine whether JMJD2D could interact with p53 protein to inhibit its DNA binding activity in liver cancer cells, we performed co-immunoprecipitation (Co-IP) assay using anti-JMJD2D and anti-p53 antibodies, respectively. As shown in Figure 5C, the interaction of endogenous JMJD2D and p53 could be found in HepG2 cells. To determine whether JMJD2D can directly bind to p53, purified recombinant p53 protein (produced by baculovirus) was incubated with *Escherichia coli*-produced GST-JMJD2D protein for GST pull-down assay. The results showed that the GST-JMJD2D protein, but not GST, was able to pull down p53 (Figure 5D), indicating that JMJD2D can directly bind to p53.

Since JMJD2D can directly bind to p53, we wondered whether binding of JMJD2D to p53 is sufficient to inhibit the p53 DNA binding activity. To do that, purified recombinant p53 protein (produced by baculovirus) and JMJD2D protein (produced by an E. *coli* extract-based cell-free protein synthesis system) were used for EMSA assay *in vitro*. The results showed that recombinant p53 protein could bind to both p21 and PUMA probes without JMJD2D addition (Figure 5E), but JMJD2D protein addition abolished the binding of p53 to the p21 and PUMA probes (Figure 5E). These results suggest that the interaction of JMJD2D with p53 alone is sufficient to inhibit the p53 DNA binding activity.

To determine how JMJD2D inhibits the p53 DNA binding activity, we examined which domains are required for the interaction between JMJD2D and p53. We expressed the different domains of JMJD2D or p53 as FLAG- or MYC-tagged proteins in HEK293T and then performed Co-IP assay. As shown in Figure 5F, the C-terminal domain (amino acids 313-523) of JMJD2D was responsible for its interaction with p53, whereas the DNA binding domain (DBD, amino acids 101-300) of p53 was responsible for its binding to JMJD2D (Figure 5G). These results implicate that JMJD2D binds to the DNA binding domain of p53 to prevent p53 from interaction with DNA.

### The demethylase activity of JMJD2D is dispensable for JMJD2D-mediated downregulation of p21 and PUMA expression

**Figure 6:**
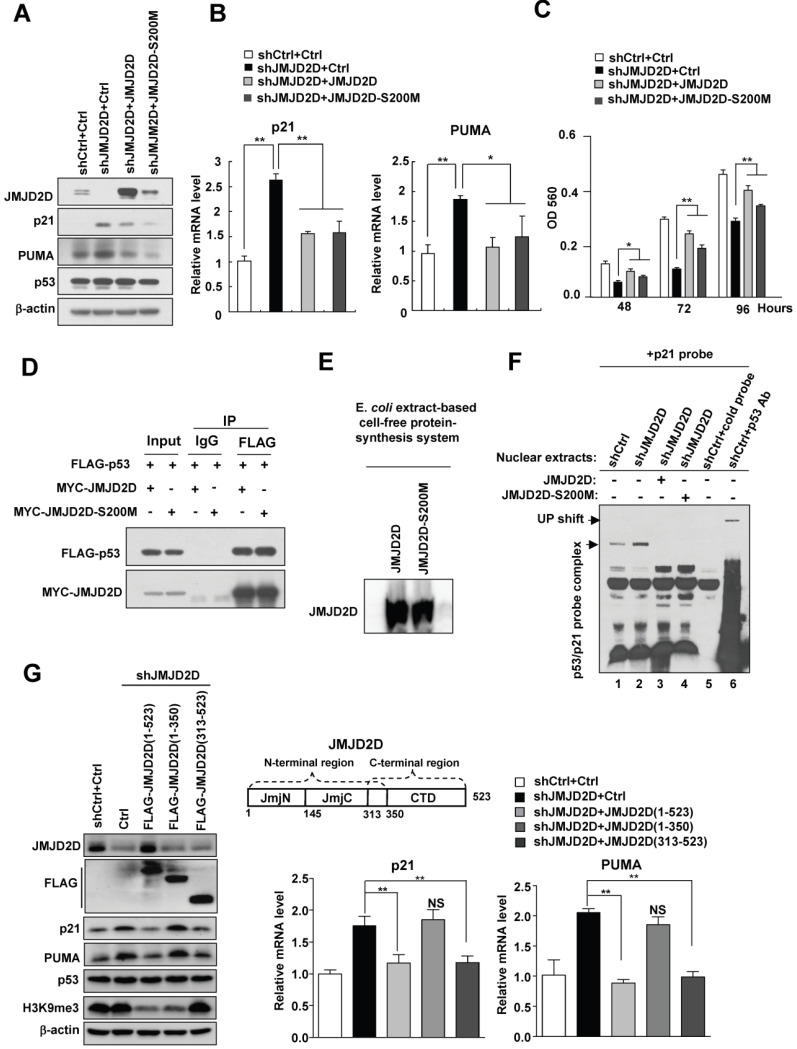
** The demethylase activity of JMJD2D is dispensable for JMJD2D-mediated downregulation of p21 and PUMA expression.** (**A-B**) Both JMJD2D and JMJD2D-S200M could inhibit the expression of p21 and PUMA in JMJD2D-knockdown HepG2 cells. Control or JMJD2D-knockdown HepG2 cells were transfected with control, JMJD2D or JMJD2D-S200M plasmids for 48 hours. Then related protein and mRNA levels were examined by Western blot and real-time PCR. (**C**) Overexpression of the JMJD2D-S200M mutant in JMJD2D-knockdown HepG2 cells partially rescued cell proliferation as determined by MTT assay. (**D**) JMJD2D and JMJD2D-S200M proteins could interact with p53 protein. The p53 plasmid together with JMJD2D or JMJD2D-S200M plasmids were transfected into 293T cells for 48 hours and the interaction between JMJD2D/JMJD2D-S200M and p53 was measured by Co-IP assay. (**E**) Wild-type JMJD2D and JMJD2D-S200M protein were produced by an E. *coli* extract-based cell-free protein synthesis system. (**F**) Both JMJD2D and JMJD2D-S200M proteins produced by an E. *coli* extract-based cell-free protein synthesis system blocked endogenous p53 binding to p21 probe. (**G**) Expression of the JMJD2D C-terminal domain (313-523) but not the N-teminal domain (1-350) in JMJD2D-knockdown HepG2 cells could inhibit the protein and mRNA expression of p21 and PUMA, respectively. n≥3, *p<0.5, **p<0.01. These experiments were performed at least three times with similar results.

It has been reported that the serine 200 of JMJD2D is essential for its histone demethylase function and mutating this serine residue in JMJD2D, from serine to methionine, generates a demethylase-dead mutant (JMJD2D-S200M)^11^, ^13^. To determine whether the demethylase activity of JMJD2D is required for the downregulation of p21 and PUMA expression, we transfected wild-type JMJD2D and JMJD2D-S200M mutant plasmids into JMJD2D-knockdown cells, respectively. As shown in Figure 6A and 6B, expression of both JMJD2D and JMJD2D-S200M in JMJD2D-knockdown cells could effectively inhibit the protein and mRNA expression of p21 and PUMA, indicating that the demethylase activity of JMJD2D is dispensable for JMJD2D-mediated downregulation of p21 and PUMA expression. Consistently, the expression of both JMJD2D and JMJD2D-S200M in JMJD2D-knockdown cells could partially restore the proliferation of JMJD2D-knockdown HepG2 cells (Figure 6C). As expected, JMJD2D and JMJD2D-S200M mutant could interact with p53 equally (Figure 6D). To determine whether the demethylase activity of JMJD2D is required for its inhibition of the p53 binding activity, wild-type JMJD2D and JMJD2D-S200M mutant protein produced by an E. *coli* extract-based cell-free protein synthesis system (Figure 6E) were added in the p53 EMSA, respectively. Both wild-type JMJD2D and JMJD2D-S200M mutant could abolish p53 binding to p21 probe (Figure 6F). These results suggest that the demethylase activity of JMJD2D is not required for JMJD2D to interact with p53 and to inhibit the p53 DNA binding activity.

Furthermore, ectopic expression of the JMJD2D C-terminal region (amino acids 313-523), which harbors the p53 interaction domain but not the demethylase catalytic JmjC domain, in JMJD2D-konckout HepG2 cells, could still effectively inhibit the protein and mRNA expression of p21 and PUMA (Figure 6G), whereas expression of the JMJD2D N-terminal region (amino acids 1-350) harboring the JmjC domain but not the p53 interaction domain could not inhibit the protein and mRNA expression of p21 and PUMA (Figure 6G). These results support the notion that the demethylase activity of JMJD2D is dispensable for JMJD2D to inhibit p21 and PUMA expression.

### JMJD2D promotes DNA replication and facilitates the formation of pre-initiative complex by inhibiting p53 signaling pathway

It has been reported that JMJD2D can promote DNA replication and facilitate the formation of pre-initiative complex 10, whereas p21 inhibits DNA replication through blocking PCNA from activating DNA polymerase δ 19. To determine whether JMJD2D promotes DNA replication through inhibiting p53 signaling pathway, EdU staining was performed. Consistent with a reduced number of JMJD2D-knockdown cells in S phase, knockdown of JMJD2D decreased the proportion of EdU-incorporated cells (Figure S9A and S9B), and p53 knockout dramatically reduced the effect of JMJD2D knockdown on the decrease of EdU-incorporated cells (Figure S9A, S9B, and S9C), suggesting that JMJD2D promotes DNA replication at least in part through inhibiting p53 signaling pathway. To determine whether JMJD2D facilitates the formation of pre-initiative complex through inhibiting p53 signaling pathway, cells were treated with mimosine to induce cell cycle arrest in G1 phase, followed by releasing into fresh medium and collecting for chromatin fractionation assay. JMJD2D knockdown retarded the chromatin binding of PCNA, Cdc45, and DNA polymerase δ (Figure S9D), whereas p53 knockout reversed the effect of JMJD2D knockdown on the chromatin binding of PCNA and DNA polymerase δ (Figure S9D; [PCNA expression: lane 17/lane 14=0.483 vs. lane 23/lane 20=0.717; lane 18/ lane 15=0.734 vs. lanes 24/ lane 21=0.775] ; [DNA polymerase δ expression: lane 17/lane14=0.550 vs. lane 23/lane 20=0.764; lane 18/ lane 15=0.600 vs. lane 24/ lane 21=0.789]), suggesting that JMJD2D facilitates the formation of pre-initiative complex at least in part through inhibiting p53 signaling pathway.

### JMJD2D deficiency protects mice against DEN-induced liver cancer initiation and progression

**Figure 7:**
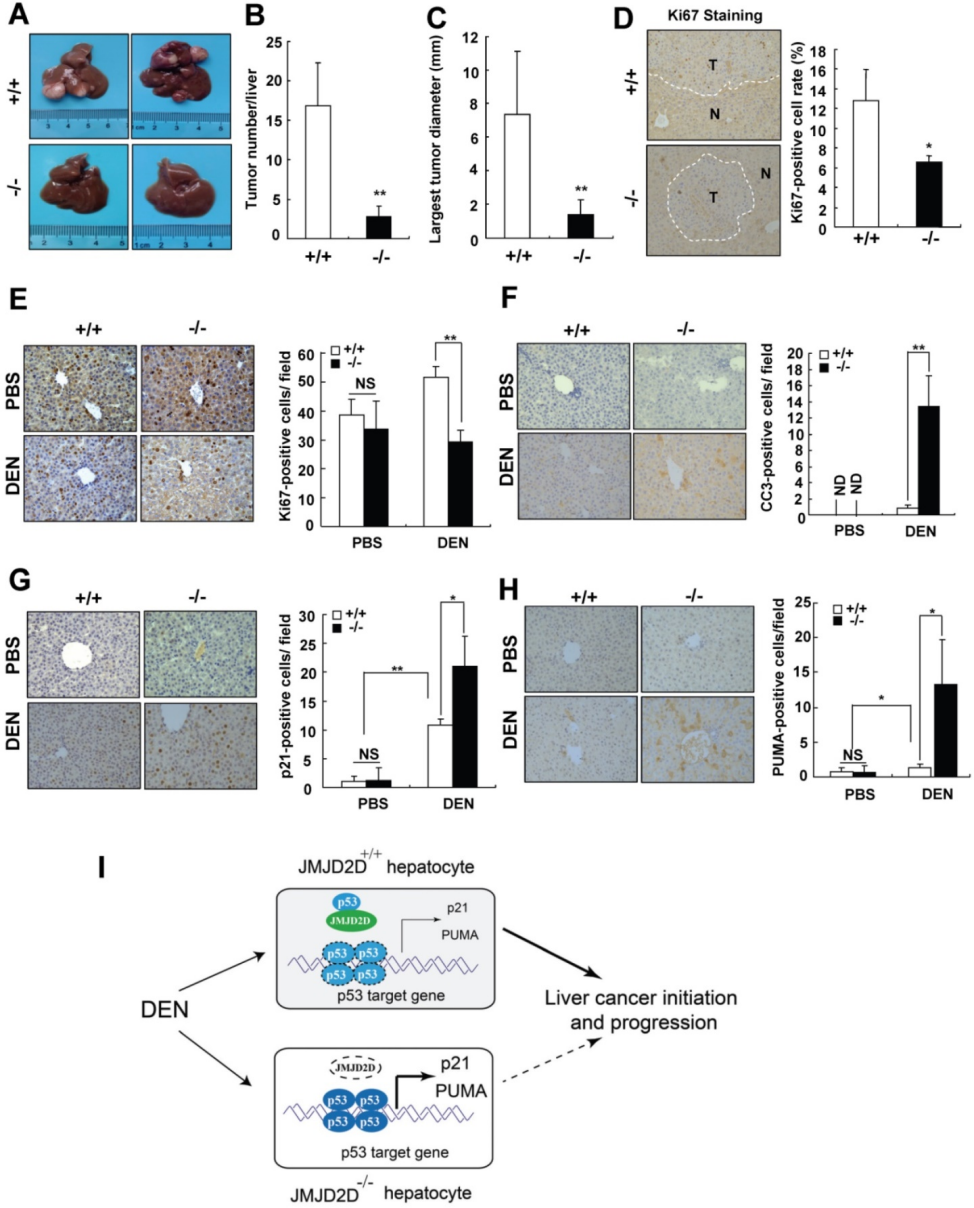
** JMJD2D deficiency protects mice against DEN-induced liver cancer initiation and progression.** (**A-C**) JMJD2D^-/-^ mice exhibited fewer and smaller liver tumors after DEN treatment. n≥5, *p<0.5, **p<0.01. (D) JMJD2D^-/-^ mice exhibited reduced Ki67-positive liver tumor cells after DEN treatment. (**E-H**) JMJD2D deficiency reduced hepatocyte proliferation, survival, and the expression of p21 and PUMA after DEN treatment. Fifteen day-old male wide-type and JMJD2D-knockout mice were intraperitoneally injected with DEN (25 mg/kg). Mice were sacrificed and livers were harvested for immunohistochemical analysis of Ki67, CC3 (Clvd caspase-3), p21, and PUMA at 48 hours post-DEN injection. n=4, *p<0.5, **p<0.01. These experiments were performed at least twice with similar results. (**I**) Schematic presentation of the mechanism by which JMJD2D promotes DEN-induced liver cancer initiation and progression by antagonizing p53. After DEN challenge, JMJD2D inhibits the expression of p21 and PUMA to promote hepatocyte proliferation and survival by antagonizing p53, consequently increasing the expansion of gene mutations, inhibiting the elimination of genotoxically-stressed cells, and resulting in liver cancer initiation and progression.

To verify the finding that JMJD2D promotes liver cancer development genetically, JMJD2D-knockout mice were used. The role of JMJD2D in primary liver cancer development was first investigated in a well-established hepatic procarcinogen diethylnitrosamine (DEN)-induced mouse liver tumor formation model. 15-day-old male wild-type and JMJD2D-knockout mice were intraperitoneally injected with DEN (25 mg/kg) and then detected the formation of tumors in the livers at 8 months post-DEN injection. As shown in Figure 7A-C, the number and size of liver tumors developed in JMJD2D^-/-^ mice were significantly decreased compared with JMJD2D^+/+^ mice. JMJD2D^-/-^ tumors exhibited a significantly lower proliferation rate compared with JMJD2D^+/+^ tumors, as determined by Ki67 staining (Figure 7D).

To further understand what makes JMJD2D^-/-^ mice resist liver cancer formation, we examined whether JMJD2D affects the response of proliferating hepatocytes to DEN at an early developmental phase of liver tumors. A single DEN dose (25 mg/kg) or the same volume of PBS (as a control) was intraperitoneally injected into 15-day-old male wild-type and JMJD2D^-/-^ mice, and then the proliferation and apoptosis of hepatocytes were examined at 48 hours later, respectively. As shown in Figure 7E and 7F, JMJD2D deficiency did not affect hepatocyte proliferation and apoptosis in the unchallenged livers, but markedly decreased hepatocyte proliferation and survival after DEN administration as determined by Ki67 and C-caspase-3 IHC staining, respectively. These results suggest that JMJD2D promotes hepatocyte proliferation and protects hepatocytes from DEN-induced apoptosis. It has been reported that p53 signaling is activated in hepatocytes after DEN treatment to eliminate genotoxically-stressed cells 20. We observed the nuclear accumulation of p-p53 (S18) in hepatocytes at 48 hours post-DEN administration (Figure S10), indicating p53 activation. Consistent with the observation that JMJD2D antagonized p53, IHC results showed that JMJD2D deficiency significantly increased the expression of p53 target genes p21 and PUMA after DEN administration (Figure 7G and 7H). These results suggest that JMJD2D deficiency inhibits hepatocyte proliferation and induces hepatocyte apoptosis by activating p53 signaling in response to DEN challenge, decreasing the expansion of gene mutations and promoting the elimination of genotoxically-stressed cells in the early developmental phase of liver tumor.

Collectively, these results suggest that JMJD2D plays an essential role in promoting DEN-induced liver cancer initiation and progression by inhibiting p53 signaling (Figure 7I).

## Discussion

In this study, we demonstrate that JMJD2D plays an essential role in promoting liver cancer initiation and progression as follows: (1) JMJD2D expression is frequently upregulated in human HCC specimens and positively correlated with a worse prognosis. (2) Knockdown of JMJD2D reduces liver cancer cell proliferation and survival, consequently reducing liver tumor growth. (3) Knockout of JMJD2D in mice significantly inhibits DEN-induced liver cancer formation and prevents hepatocyte proliferation and survival in liver cancer initiation stage.

JMJD2D promotes liver cancer initiation and progression at least in part through antagonizing the p53 tumor suppressor since inhibition of p53 activity is required for liver cancer initiation and progression 5, 20. It is well acknowledged that the cell-cycle arresting and apoptosis promoting functions of p53 are important for preventing tumor development, and p53-induced G1/S boundary cell cycle arrest and apoptosis largely (albeit not exclusively) go through direct transcriptional induction of CDK inhibitor p21 and PUMA, respectively 5, 6. It has been reported that histone methylase EZH2 and histone demethylase KDM5A can modulate p53 signaling pathway through regulating p53 expression 21, 22. However, knockdown of JMJD2D does not affect p53 protein expression, excluding the possibilities that JMJD2D regulates p53 signaling pathway through controlling the expression of p53 protein. Mechanistically, JMJD2D directly interacts with p53 and inhibits p53 binding to the promoters of its target genes such as p21 and PUMA, consequently inhibiting p21 and PUMA gene transcription. As a histone demethylase, JMJD2D is frequently recruited by the transcription factor to the gene promoter and affects gene transcriptional expression through demethylating H3K9 on the promoter 8, 13, 23, 24. Similarly, JMJD2D may affect the activities of some proteins by demethylating the lysine residues. Intriguingly, we found that the demethylase activity of JMJD2D is not required for JMJD2D to interact with p53 and inhibit p53-induced p21 and PUMA expression by using the demethylase-dead JMJD2D mutant (JMJD2D-S200M), excluding the possibility that JMJD2D inhibits p53 DNA binding ability through demethylating the lysine residues of p53. Therefore, JMJD2D has a demehylase-independent function as a p53 antagonist to promote liver cancer progression. JMJD2D interacts with p53 at its DNA binding domain, and the demethylase activity of JMJD2D is not required for their interaction, suggesting that JMJD2D interacts with the DNA binding domain of p53 to prevent p53 from binding to the promoters of p53 target genes via this domain physically, resulting in disrupting p53-mediated transcription. Given the important role of p53 in genome surveillance and elimination of genotoxically stressed cells 5, 6, 20, our study also suggests that inhibition of p53 activity by JMJD2D plays a key role in the early step of liver cancer initiation before p53-inactivating mutations happen.

Given that JMJD2D can interact with p53 and inhibit p53 recruitment to its target gene promoters, we speculated that JMJD2D may negatively regulate the transcription of a cohort of p53 target genes related to cell proliferation and apoptosis, and the degree to which JMJD2D regulates p53 target genes depends on the intrinsic effect of p53 on these genes. Indeed, in addition to p21 and PUMA, knockdown of JMJD2D could promote the transcription of p53 target gene NOXA in both HepG2 and SK-Hep1 cells (Figure S11A and S11B), whereas knockdown of JMJD2D only promoted the expression of GADD45a and Sestrin2 (another two p53 target genes) in HepG2 cells (Figure S11A), but not in SK-hep1 cells (Figure S11B). It has been reported that NOXA can induce apoptosis 5, 25.

Therefore, upregulation of NOXA in JMJD2D-knockdown cells may also contribute to the sensitivity of liver cancer cells to chemotherapeutic drug-induced apoptosis. Knockdown of p53 decreased the mRNA levels of p21, PUMA, and NOXA in both HepG2 and SK-Hep1 cells (Figure S11C and S11D), whereas knockdown of p53 decreased the mRNA levels of GADD45a and Sestrin2 in HepG2 cells (Figure S11C), but not in SK-Hep1 cells (Figure S11D). These results suggest that the effect of p53 on the expression of its target genes is cell-context dependent and JMJD2D blocks the expression of p53 target genes that are positively regulated by endogenous p53. Knockdown of JMJD2D did not affect the expression of p21 and PUMA in human liver cancer Huh-7 cells (Figure S5), which express the p53-Y220C mutant with impaired transactivation activity 16-18, supporting the notion that the effect of JMJD2D on the expression of p53 target genes depends on the intrinsic transcriptional function of p53.

Somatic p53 mutations are one of the most frequent alterations in human cancers, and most mutations are missense single-base substitution in coding sequence 4, 26, 27. It has been reported that somatic mutation rate of p53 gene in liver cancer is about 31.9%^4^, indicating that a large number of liver tumors still retain wild-type p53 expression, but the abundant or transcriptional activity of wild-type p53 may be reduced by some mechanisms. For example, E3 ubiquitin ligase MDM2 is frequently upregulated in liver tumor to rapidly degrade p53 28, 29 . In this study, we reveal a novel mechanism for inhibition of p53 transcriptional activity in liver tumor: JMJD2D interacts with p53 to physically prevent p53 binding to the promoter of its target gene. Given the powerful anti-cancer function of p53, restoring the expression or transcriptional activity of wild-type p53 becomes an attractive strategy for cancer therapy. Indeed, several novel potent MDM2 inhibitors that inhibit MDM2-p53 interaction are under development or in clinical trials 30, 31. Therefore, the development of JMJD2D inhibitors that can downregulate JMJD2D expression or block the interaction between JMJD2D and p53 may represent a novel strategy to restore the transcriptional activity of wild-type p53 for liver cancer treatment.

We observed that knockout of p53 significantly impaired the effects of JMJD2D knockdown on liver cancer cell proliferation and apoptosis, but knockdown JMJD2D could still partially inhibit cell proliferation of p53-KO cells, whereas overexpression of JMJD2D could promote the proliferation of p53-KO cells (Figure S12), suggesting that apart from p53 signal pathway, JMJD2D regulates liver cancer cell proliferation through other signal pathways. Our previous study showed that JMJD2D promoted colorectal cancer progression by enhancing Wnt signaling via inducing β-catenin expression and serving as a coactivator for β-catenin 13. Similarly, we found that knockdown of JMJD2D significantly inhibited the protein and mRNA expression of β-catenin and its target gene c-Myc in HepG2 cells (Figure S13A and S13B). Promoter reporter assay showed that knockdown of JMJD2D decreased promoter activities of c-Myc and Topflash (Figure S13C and S13D), suggesting that JMJD2D enhances the transcription of c-Myc through activating Wnt/β-catenin signaling in HepG2 cells. Rescuing β-catenin expression in JMJD2D-knockdown HepG2 cells could partially restore c-Myc expression and cell proliferation (Figure S13E and S13F), suggesting that JMJD2D can also promote liver cancer cell proliferation through enhancing Wnt/β-catenin signaling. The JMJD2D-S200M mutant failed to promote the expression of β-catenin and c-Myc (Figure S13G), suggesting that the demethylase activity of JMJD2D is required for its function for inducing the expression of β-catenin and c-Myc. Overexpression of JMJD2D could increase the expression of β-catenin and c-Myc in both p53-WT and p53-KO cells (Figure S14), suggesting that JMJD2D-induced β-catenin and c-Myc expression is independent of p53 and also contributes to JMJD2D-induced proliferation of p53-KO cells. Furthermore, we found that JMJD2D expression was positively correlated with β-catenin in human liver cancer specimens from a publicly available TCGA data (Figure S15A). However, no correlation was established between the expression levels of JMJD2D and c-Myc in the same data set (Figure S15B). It has been reported that overexpression of c-Myc can activate p53 via ATM (ataxiateangiectasia mutated kinase) signaling pathway to induce cell apoptosis 32. Therefore, the function of JMJD2D as a p53 antagonist may reduce the apoptotic side effect of JMJD2D-induced activation of oncogenic signaling pathway during liver cancer initiation and progression.

In this study, we found that overexpression of the JMJD2D N-terminal region (amino acids 1-350) harboring the demethylase catalytic JmjC domain but not the p53 interaction domain in JMJD2D-knockdown HepG2 cells could induce the expression of β-catenin and c-Myc (Figure S16A), but could not inhibit the expression of p21 and PUMA (Figure 6G), whereas overexpression of the JMJD2D C-terminal region (amino acids 313-523) harboring the p53 interaction domain but not the JmjC domain could inhibit the expression of p21 and PUMA(Figure 6G), but could not affect the expression of β-catenin and c-Myc (Figure S16A); Overexpression of either the JMJD2D N-terminal region or the C-terminal region in JMJD2D-knockdown HepG2 cells could partially restore cell proliferation (Figure S16B). Based on these results, we conclude that both the p53 interaction ability and the histone demethylase activity of JMJD2D contribute to its oncogenic function in liver cancer. They work independently through inhibiting p53 signaling pathway and activating Wnt/β-catenin signaling pathway, respectively.

Given that the effect of JMJD2D on the expression of p53 target genes depends on the intrinsic transcriptional function of p53 and some mutated p53 proteins loss their functions as the transcription factors, we speculated that JMJD2D may be more important for promoting the progression of p53 wild-type liver tumors than p53-mutant liver tumors in which JMJD2D does not downregulate the expression of p53 target genes such as p21 and PUMA. For example, JMJD2D knockdown increased the protein and mRNA expression of p21 and PUMA in HepG2 cells expressing the p53 wild-type proteins, but not in Huh-7 cells (Figure S17A), which express the mutant (Y220C) p53 proteins. Ectopic expression of the JMJD2D C-terminal region, which harbors the p53 interaction domain, could inhibit the expression of p21 and PUMA and partially restore the proliferation of JMJD2D-knockdown HepG2 cells, but did not inhibit the expression of p21 and PUMA (Figure S17A) and failed to restore the proliferation of JMJD2D-knockdown Huh-7 cells (Figure S17B). Ectopic expression of the JMJD2D N-terminal region harboring the demethylase catalytic JmjC domain could induce the expression of β-catenin and c-Myc in both JMJD2D-knockdown HepG2 (Figure S16A) and Huh-7 cells (Figure S17A), and partially restore the proliferation of JMJD2D-knockdown HepG2 (Figure S16B) and Huh-7 cells (Figure S17B). Collectively, these results suggest that JMJD2D promotes p53 wild-type liver tumors through inhibiting p53 signaling pathway and activating Wnt/β-catenin signaling pathway simultaneously, whereas JMJD2D promotes p53-mutant liver tumors through activating Wnt/β-catenin signaling pathway.

A previous study by Janknecht et al. shows that JMJD2D has an oncogenic role in cancer and binds p53 through similar domains identified in our current study 33. Intriguingly, their study concludes that JMJD2D cooperates with p53 to induce the expression of cell cycle inhibitor p21 as demonstrated by the observations that combination of p53 and JMJD2D synergistically activate the p21 promoter reporter in HEK293T cells and overexpression of JMJD2D promotes p21 protein expression in U2OS osteosarcoma cells 33. Clearly, their conclusion that JMJD2D cooperates with p53 to induce the expression of cell cycle inhibitor p21 cannot support the oncogenic role of JMJD2D in cancer, whereas the conclusion of our current study that JMJD2D antagonizes p53 to inhibit p21 expression can support the oncogenic role of JMJD2D in cancer. One possible reason for the apparent discrepancies is that the experiments for studying the relationship among JMJD2D, p53, and p21 by Janknecht et al. are only performed in the systems of p21 promoter reporter assay in HEK293T cells and ectopic overexpression of JMJD2D in U2OS cells, which may generate some artificial effects not to reflect the natural relationship among JMJD2D, p53, and p21 in cancer cells. The other possible reason for the discrepancies may be cell-context dependent since the cells used by Janknecht et al. (HEK293T and U2OS cells) are different from ours (HepG2 and SK-Hep1 cells).

In summary, our study demonstrates that JMJD2D can antagonize the p53 tumor suppressor and activate an oncogenic signaling pathway (such as Wnt/β-catenin signaling pathway) simultaneously to drive liver tumorigenesis, suggesting that JMJD2D may serve as a novel target for liver cancer treatment.

## Supplementary Material

Supplementary figures.

## Abbreviations

JMJD2D: Jumonji C domain-containing histone demethylase protein 2D
p21: cyclin-dependent kinase inhibitor p21
PUMA: p53 upregulated modulator of apoptosis
PCNA: proliferating cell nuclear antigen
MTT: 3-(4,5-dimethyl-2-thiazolyl)-2,5-diphenyl-2-H-tetrazolium
HCC: hepatocellular carcinoma
DEN: diethylnitrosamine
