# Supplementary material for: Demethylase-independent function of JMJD2D as a novel antagonist of p53 to promote Liver Cancer initiation and progression
Source: Theranostics. 2020 Jul 11;10(19):8863–79. doi: 10.7150/thno.45581 (PMC7392006; doi:10.7150/thno.45581)
Supplement: Supplementary file 1 — Supplementary figures. [file thnov10p8863s1.pdf]

Supplementary Figure 1

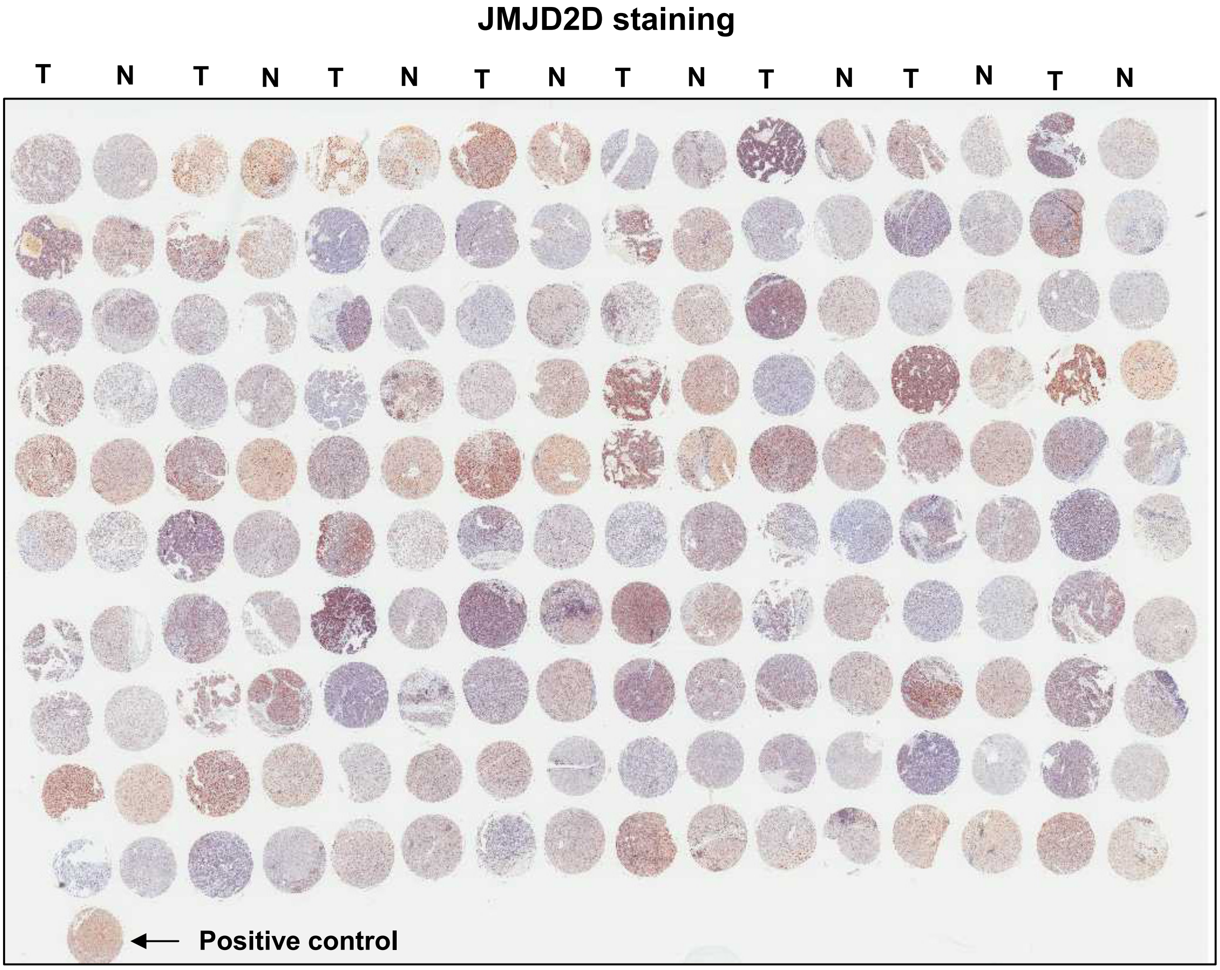

Figure S1. JMJD2D protein expression is frequently upregulated in human HCC specimens compared with non-tumorous liver tissues. T: tumor; N: non-tumorous tissue.

Supplementary Figure 2

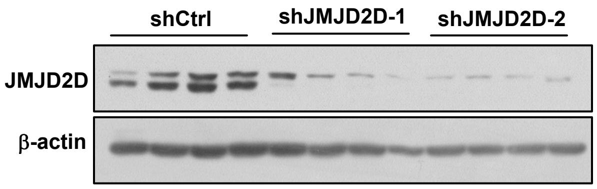

Figure S2. The protein levels of JMJD2D in JMJD2D-knockdown xenograft tumors were significantly lower than those in control tumors. JMJD2D protein levels were detected in control and JMJD2D-knockdown xenograft tumors by Western blot. These experiments were performed twice with similar results.

Supplementary Figure 3

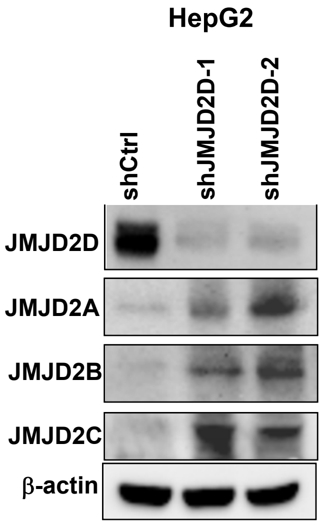

Figure S3 Compensatory upregulation of JMJD2A, JMJD2B, and JMJD2C proteins is detected in JMJD2D-knockdown HepG2 cells by Western blot. These experiments were performed twice with similar results.

Supplementary Figure 4

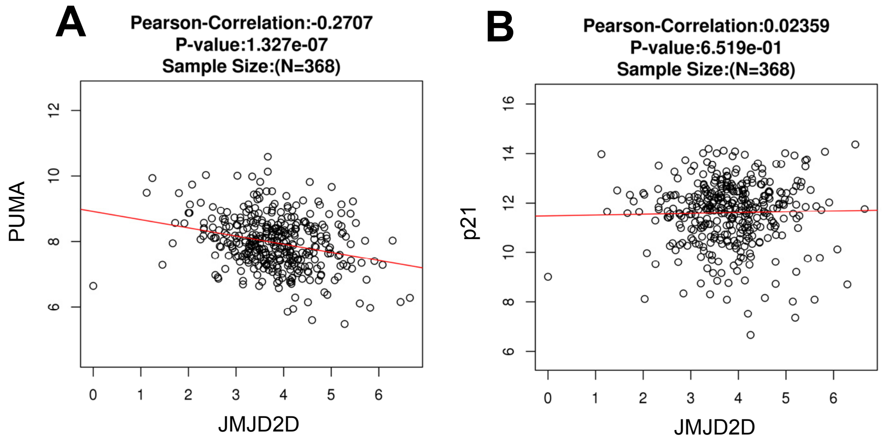

Figure S4. Correlation analysis of the expression of JMJD2D with PUMA or p21 in TCGA data set.（A）JMJD2D expression was inversely correlated with the expression of PUMA in human liver cancer specimens. (B) No correlation was established between the expression of JMJD2D and p21 in human liver cancer specimens. These correlations were analyzed using LinkedOmics website in the same TCGA data set.

Supplementary Figure 5

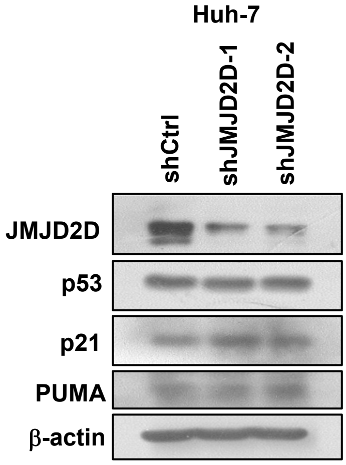

Figure S5. Knockdown of JMJD2D does not affect the expression of p21 and PUMA in human liver cancer Huh-7 cells. The protein expression levels of p21 and PUMA were detected by Western blot analysis in control and JMJD2D-knockdown Huh-7 cells. These experiments were performed twice with similar results.

Supplementary Figure 6

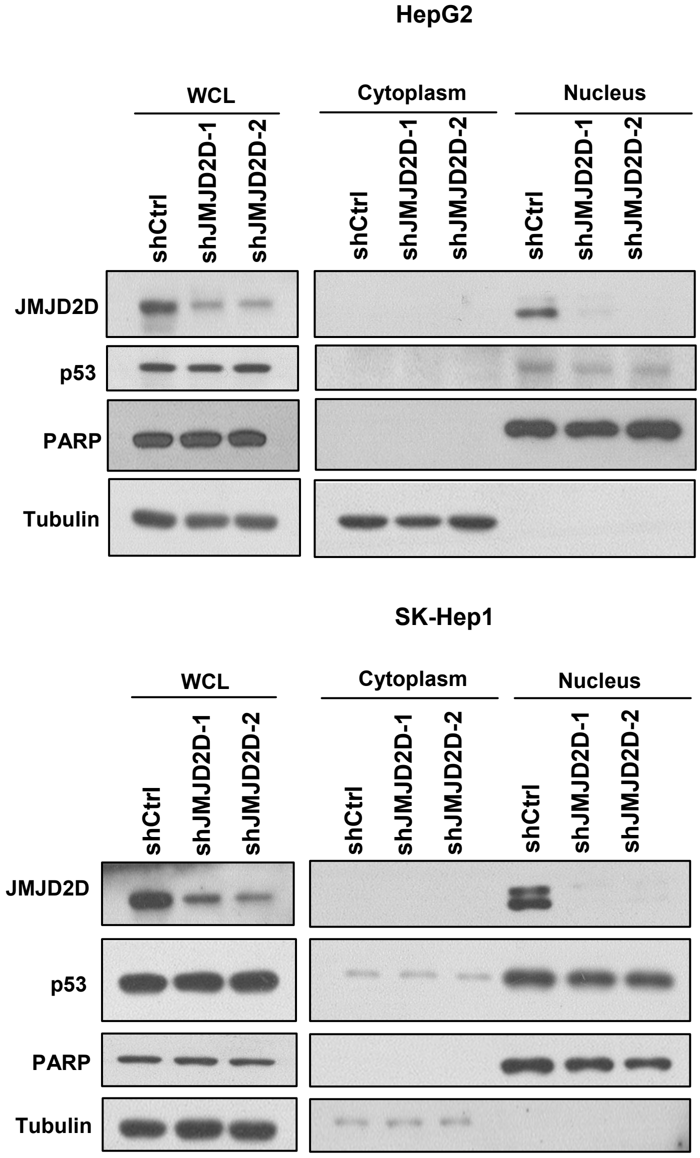

Figure S6. Knockdown of JMJD2D does not affect p53 protein levels in both cytoplasm and nucleus. SK-Hep1 or HepG2 cells fractionation was carried out and fractions were analyzed by Western blot. These experiments were performed at least three times with similar results.

Supplementary Figure 7

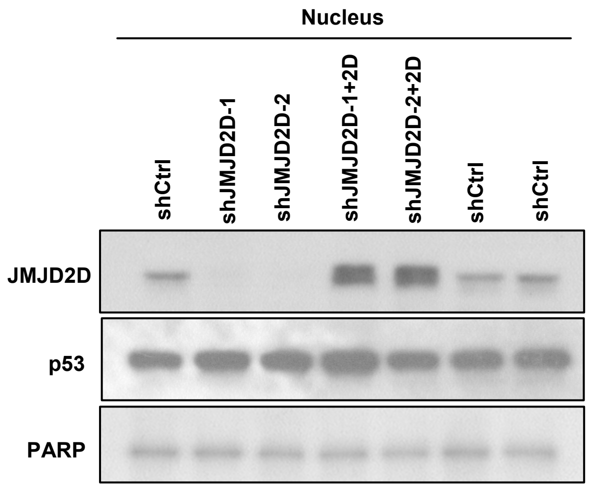

Figure S7. One μg of total nuclear protein of SK-Hep1 cells is used to perform Western blot as the protein input of EMSA in Figure 5B.

Supplementary Figure 8

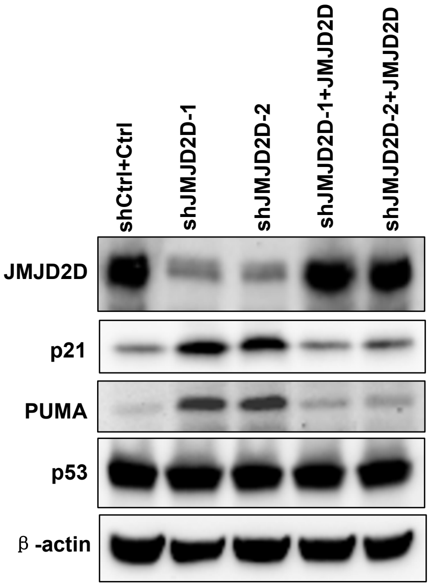

Figure S8. Restoration of JMJD2D expression in JMJD2D-knockdown cells dramatically reduced the upregulation effects of JMJD2D knockdown on the expression of p21 and PUMA protein in SK-Hep1 cells .

Supplementary Figure 9

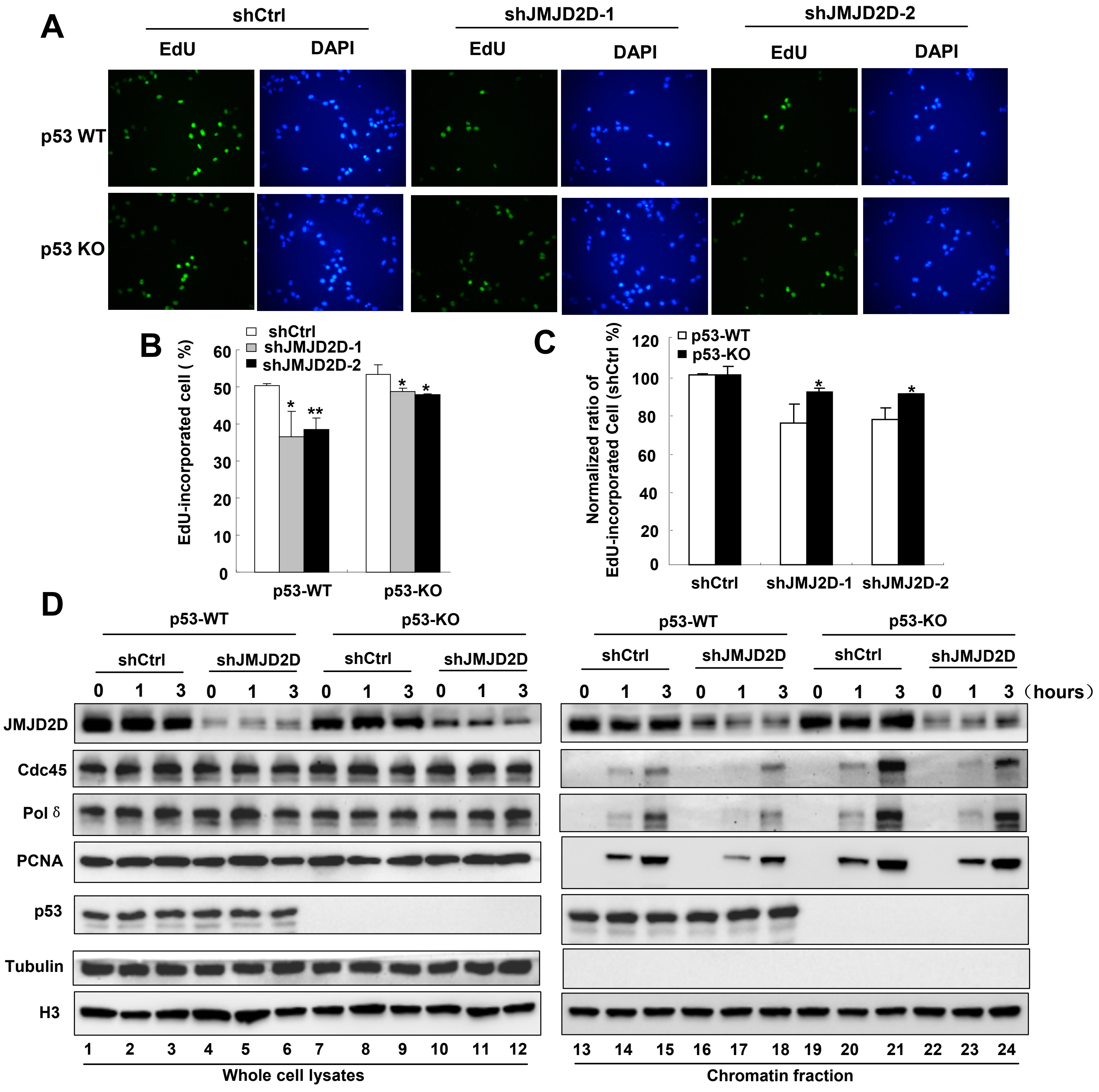

Figure S9. JMJD2D promotes DNA replication and facilitates the formation of pre-initiative complex by inhibiting p53 signaling pathway. （A,B,C) JMJD2D promotes DNA replication at least in part through inhibiting p53 signaling pathway. DNA synthesis was measured by EdU staining. EdU staining was performed according to the manufacturer's instructions (Invitrogen, Carlsbad, CA, USA). *p<0.05, **p<0.01. (D) JMJD2D facilitates the formation of pre-initiative complex at least in part through inhibiting p53 signaling pathway. Cells were treated with 10 mM mimosine for 24 hours to induce cell cycle arrest in G1 phase, followed by releasing into fresh medium for indicated time points and collecting for chromatin fractionation assay. These experiments were performed at least twice with similar results.

Supplementary Figure 10

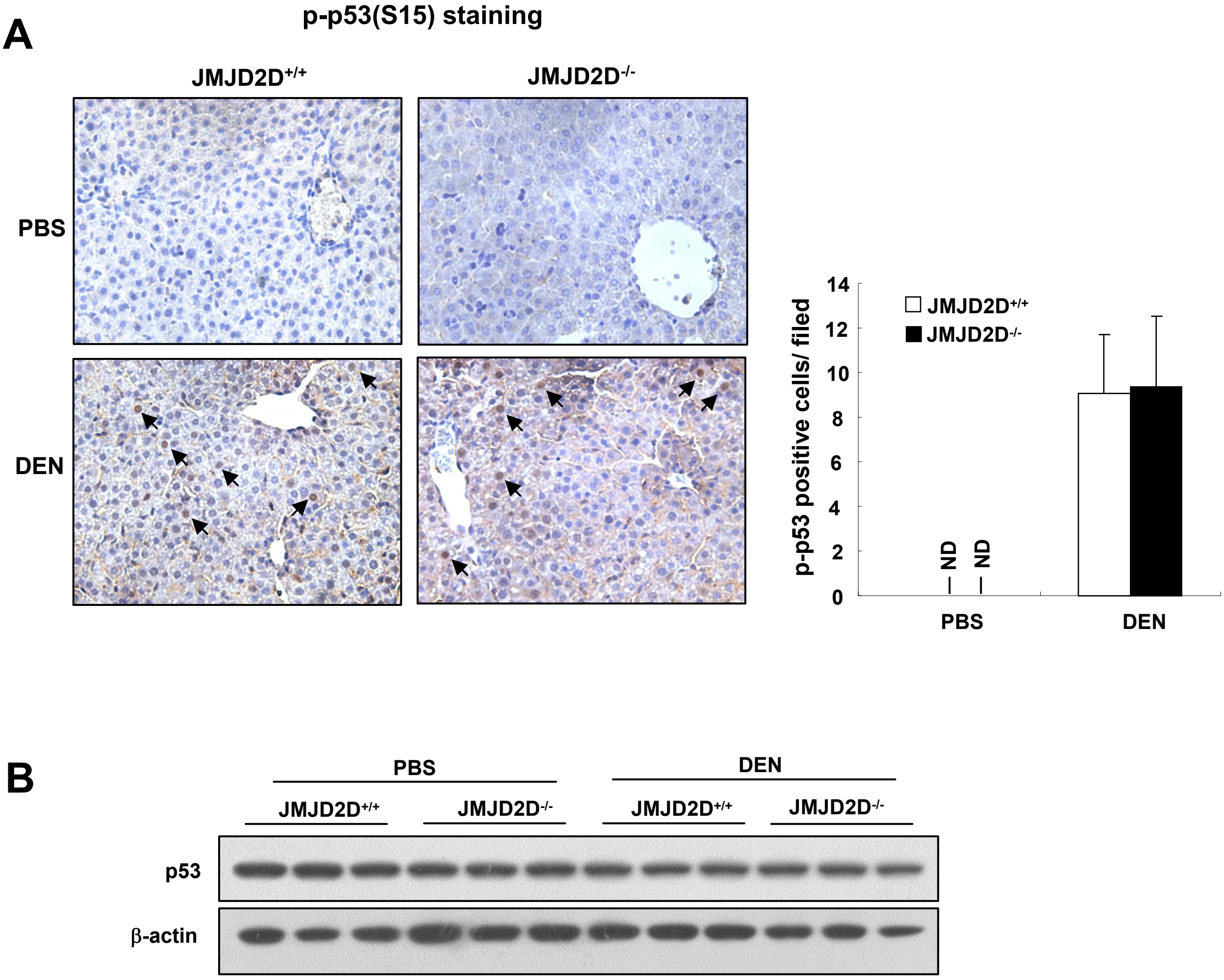

Figure S10. Nuclear accumulation of p-p53 (S18) in hepatocytes at 48 hours post-DEN administration. (A,B). Fifteen-day-old male wide-type and JMJD2D-knockout mice were intraperitoneally injected with DEN (25 mg/kg). Mice were sacrificed and the livers were harvested at 48 hours post-DEN injection. Expression of p-p53 and p53 was detected by immunohistochemical and Western blot analysis, respectively. These experiments were performed twice with similar results.

Supplementary Figure 11

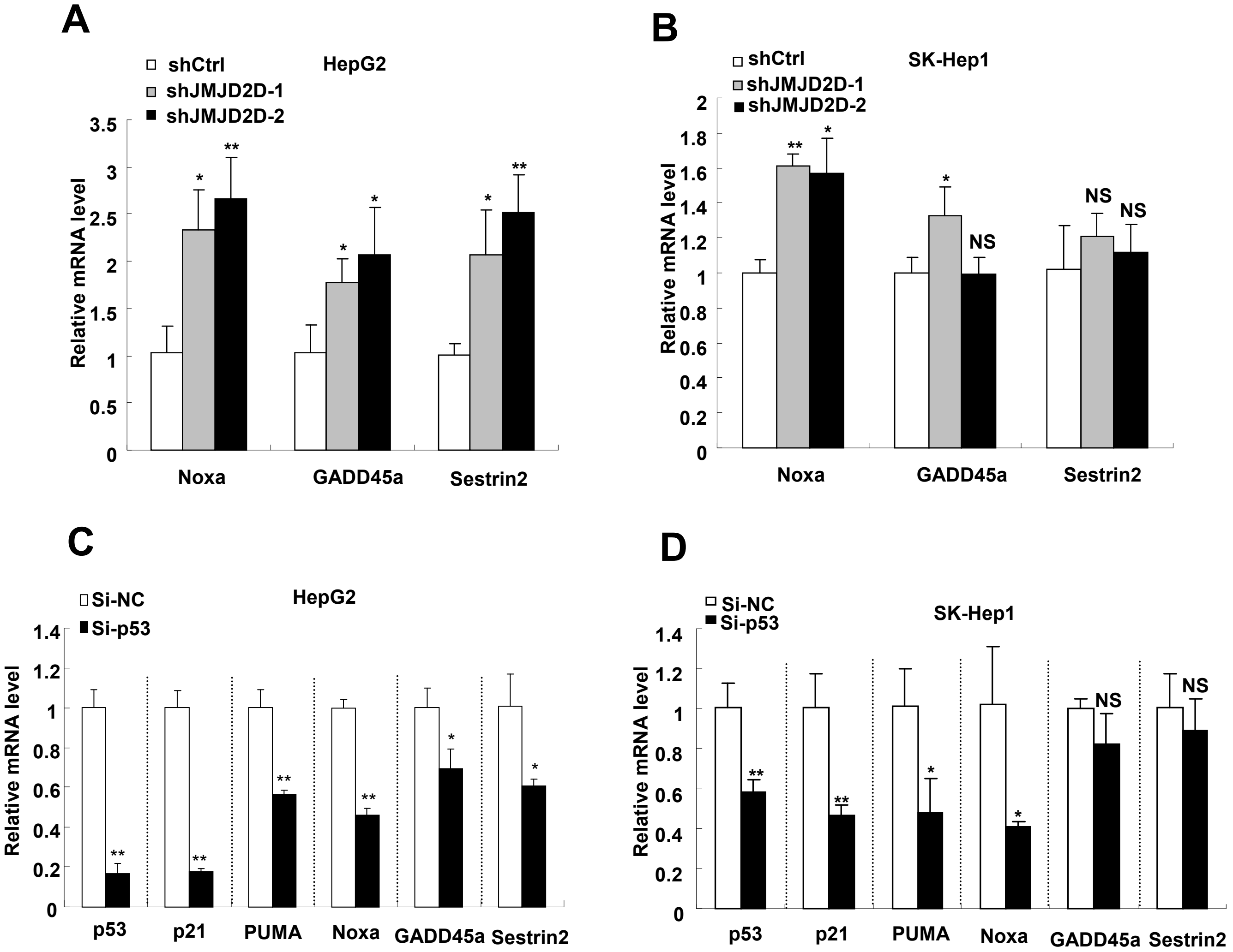

Figure S11. The effects of JMJD2D knockdown on the expression of p53 target genes.（A）Knockdown of JMJD2D increased the mRNA expression levels of NOXA,GADD45a and Sestrin2 in HepG2 cells. (B) Knockdown of JMJD2D increased the mRNA expression levels of NOXA, but did not affect GADD45a and Sestrin2 expression levels in SK-Hep1 cells. (C) Knockdown of p53 decreased the mRNA expression levels of p53, p21, PUMA, NOXA, GADD45a and Sestrin2 in HepG2 cells. (D) Knockdown of p53 decreased the mRNA expression levels of p53, p21, PUMA and NOXA, but did not affect GADD45a and Sestrin2 expression levels in SK-Hep1 cells . *$p<0.05$; **$p<0.01$. These experiments were performed at least twice with similar results.

Supplementary Figure 12

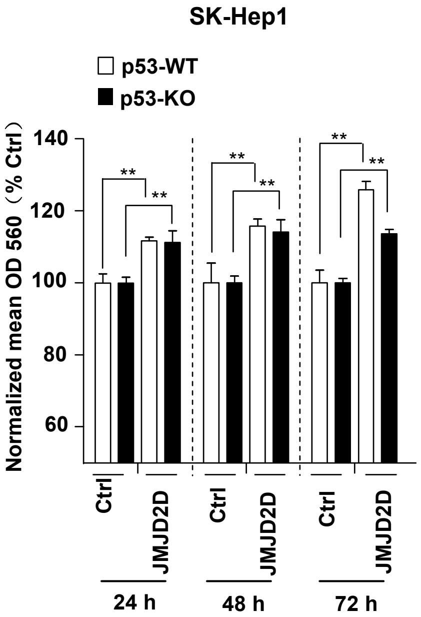

Figure S12. Overexpression of JMJD2D promotes the proliferation of p53-WT and p53-KO SK-Hep-1 cells *p<0.05, **p<0.01. These experiments were performed twice with similar results.

Supplementary Figure 13

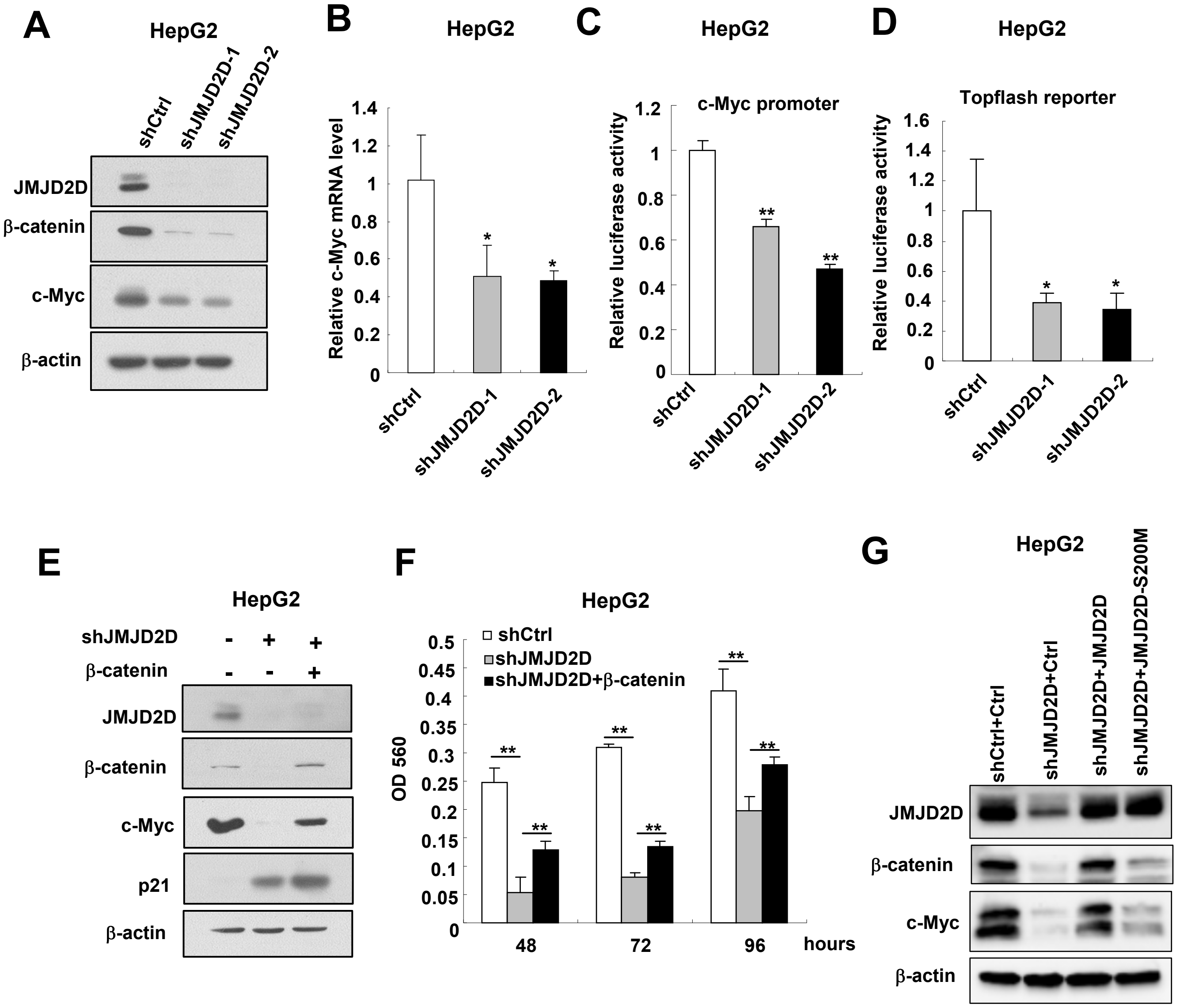

Figure S13. JMJD2D promotes liver cancer progression by enhancing Wnt/β-catenin signaling. (A) Knockdown of JMJD2D decreased β-catenin and c-Myc protein expression. (B) Knockdown of JMJD2D decreased c-Myc mRNA expression. (C,D) Knockdown of JMJD2D decreased c-Myc promoter and Topflash reporter activities, respectively.（E）Rescued expression of β-catenin in JMJD2D-knockdown HepG2 cells increased c-Myc expression. (F) Rescued expression of β-catenin in JMJD2D-knockdown HepG2 cells increased cell proliferation. (G) Overexpression of the JMJD2D-S200M mutant in JMJD2D-knockdown cells failed to promote the expression of β-catenin and c-Myc as compared to wild-type JMJD2D. *p<0.05, **p<0.01. These experiments were performed at least three times with similar results.

Supplementary Figure 14

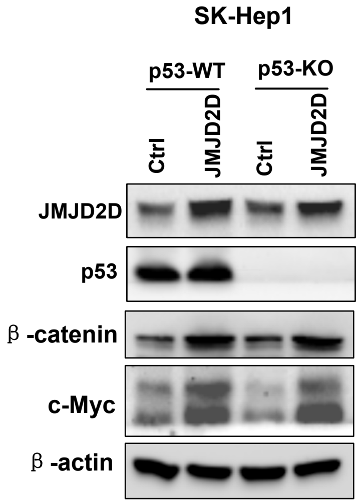

Figure S14. Overexpression of JMJD2D increases the expression of β-catenin and c-Myc in both p53-WT and p53-KO SK-Hep1 cells. These experiments were performed twice with similar results.

Supplementary Figure 15

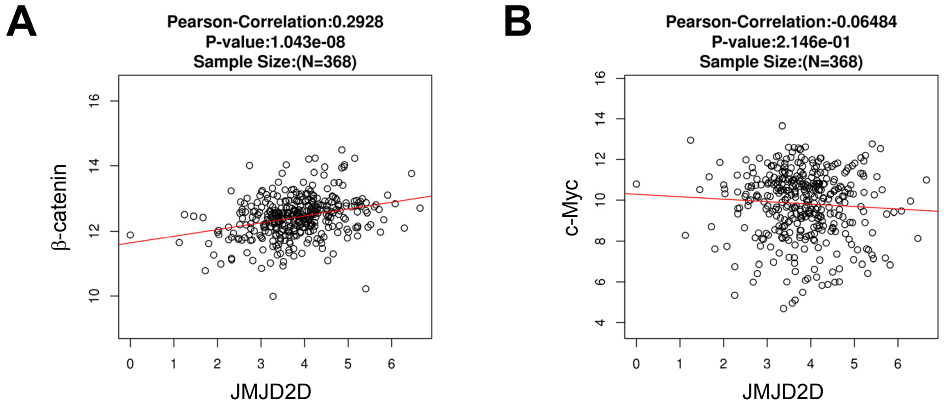

Figure S15. Correlation analysis of the expression of JMJD2D and β-catenin or c-Myc in TCGA data set. （A） JMJD2D expression was positively correlated with β-catenin in human liver cancer specimens in TCGA data.（B） No correlation was established between the expression levels of JMJD2D and c-Myc in human liver cancer specimens in TCGA data.

Supplementary Figure 16

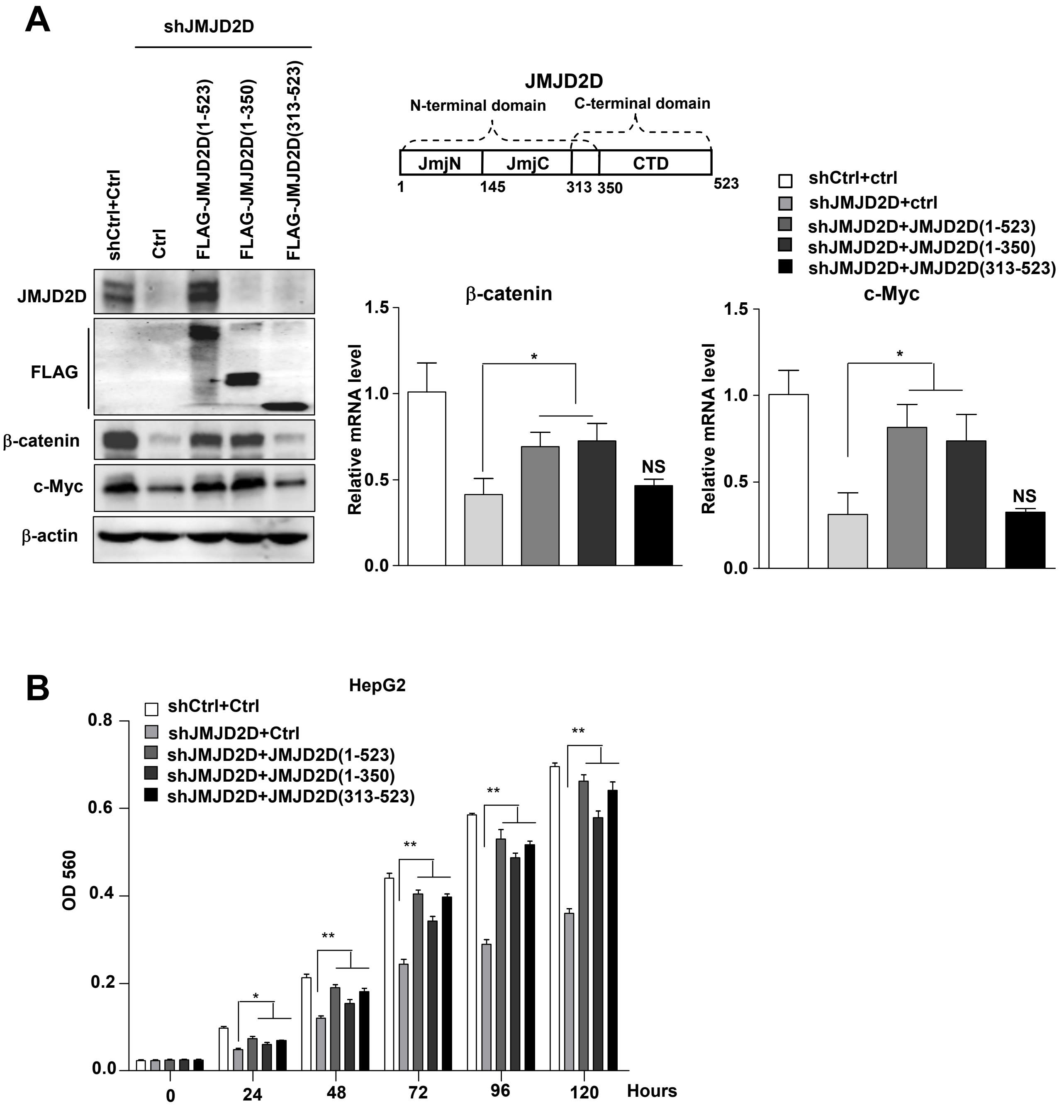

Figure S16. Effects of the expression of the JMJD2D N-terminal region or the C-terminal region in JMJD2D-knockdown cells on β-catenin and c-Myc expression as well as on cell proliferation.（A）Expression of the JMJD2D N-terminal region (1-350) but not the C-terminal region (313-523) in JMJD2D-knockdown cells restored the protein and mRNA expression of β-catenin and c-Myc in HepG2 cells. (B) Expression of either the N-terminal region or the C-terminal region in JMJD2D-knockdown cells could partially restore cell proliferation as determined by MTT assays in HepG2 cells. *p<0.05; **p<0.01. These experiments were performed at least three times with similar results.

Supplementary Figure 17

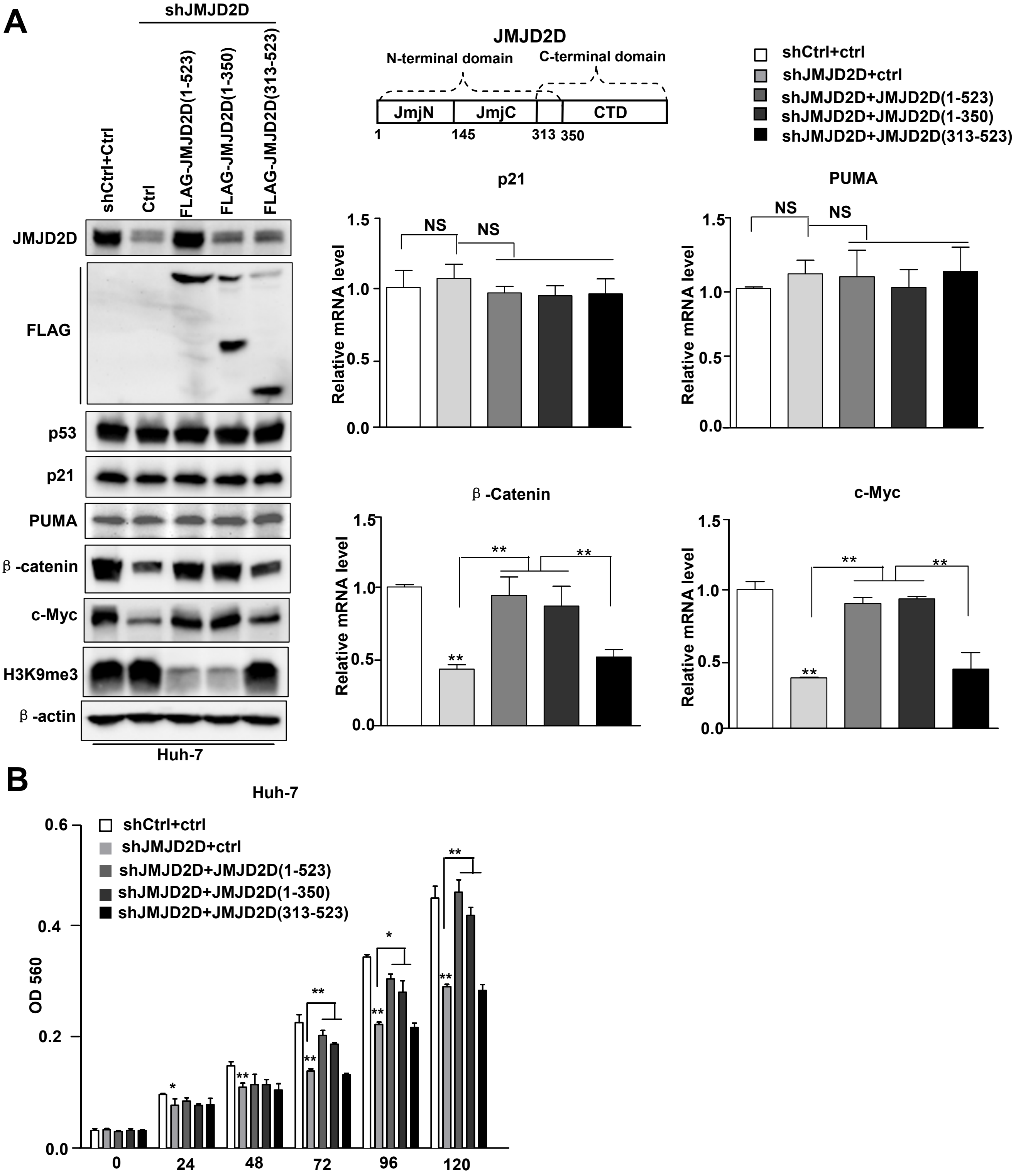

Figure S17. The effects of the ectopic expression of JMJD2D N-terminal region or the C-terminal region in JMJD2D-knockdown cells on the expression of p21, PUMA, β-catenin and c-Myc as well as on the proliferation of Huh-7 cells. （A） Expression of the JMJD2D N-terminal region (1-350) in JMJD2D-knockdown cells restored the protein and mRNA expression of β-catenin and c-Myc. Expression of the C-terminal region (313-523) did not affect the protein and mRNA expression of p21 and PUMA in Huh-7 cells. (B) Expression of the N-terminal region but not the C-terminal region in JMJD2D-knockdown cells could partially restore cell proliferation as determined by MTT assays. *p<0.05; **p<0.01. These experiments were performed at twice times with similar results.
